# Posicionamento sobre Hipertensão Arterial e Espiritualidade – 2021

**DOI:** 10.36660/abc.20210723

**Published:** 2021-09-01

**Authors:** Fernando Nobre, Roberto Esporcatte, Andréa Araujo Brandão, Álvaro Avezum, Audes Diógenes Magalhães Feitosa, Celso Amodeo, Eduardo Costa Duarte Barbosa, Emilio Hideyuki Moriguchi, Fernando Antônio Lucchese, Hermilo Borba Griz, José Carlos Nicolau, Lucélia Batista Neves Cunha Magalhães, Marco Antônio Mota-Gomes, Mario Henrique Elesbão de Borba, Mauro Ricardo Nunes Pontes, Paulo César Brandão Veiga Jardim, Pedro Pimenta de Mello Spineti, Ricardo Mourilhe-Rocha, Roberto Dischinger Miranda, Sérgio Lívio Menezes Couceiro, Weimar Kunz Sebba Barroso

**Affiliations:** 1 Universidade de São Paulo Faculdade de Medicina de Ribeirão Preto Ribeirão Preto SP Brasil Hospital das Clínicas da Faculdade de Medicina de Ribeirão Preto da Universidade de São Paulo, Ribeirão Preto, SP – Brasil; 2 Hospital São Francisco Ribeirão Preto SP Brasil Hospital São Francisco, Ribeirão Preto, SP – Brasil; 3 Universidade do Estado do Rio de Janeiro Rio de Janeiro RJ Brasil Universidade do Estado do Rio de Janeiro (UERJ), Rio de Janeiro, RJ – Brasil; 4 Hospital Pró-Cradíaco Rio de Janeiro RJ Brasil Hospital Pró-Cradíaco, Rio de Janeiro, RJ – Brasil; 5 Hospital Alemão Oswaldo Cruz São Paulo SP Brasil Hospital Alemão Oswaldo Cruz, São Paulo, SP – Brasil; 6 Universidade Federal de Pernambuco Recife PE Brasil Universidade Federal de Pernambuco, Recife, PE – Brasil; 7 Pronto Socorro Cardiológico de Pernambuco Recife PE Brasil Pronto Socorro Cardiológico de Pernambuco (PROCAPE), Recife, PE – Brasil; 8 Universidade Federal de São Paulo Recife SP Brasil Universidade Federal de São Paulo (UNIFESP), São Paulo, SP – Brasil; 9 Santa Casa de Misericórdia de Porto Alegre Porto Alegre RS Brasil Santa Casa de Misericórdia de Porto Alegre, Porto Alegre, RS – Brasil; 10 Universidade Federal do Rio Grande do Sul Porto Alegre RS Brasil Universidade Federal do Rio Grande do Sul, Porto Alegre, RS – Brasil; 11 Hospital Agamenon Magalhães Recife PE Brasil Hospital Agamenon Magalhães, Recife, PE – Brasil; 12 Universidade de São Paulo Faculdade de Medicina Instituto do Coração (InCor), Hospital das Clínicas São Paulo SP Brasil Instituto do Coração (InCor), Hospital das Clínicas da Faculdade de Medicina da Universidade de São Paulo (HCFMUSP), São Paulo, SP – Brasil; 13 Centro Universitário de Tecnologia e Ciência Salvador BA Brasil Centro Universitário de Tecnologia e Ciência (UniFTC), Salvador, BA – Brasil; 14 Universitário CESMAC Maceió AL Brasil Centro Universitário CESMAC, Maceió, AL – Brasil; 15 Cardio Clínica do Vale Lajeado RS Brasil Cardio Clínica do Vale, Lajeado, RS – Brasil; 16 Liga de Hipertensão Arterial Goiânia GO Brasil Liga de Hipertensão Arterial, Goiânia, GO – Brasil; 17 Hospital do Coração de Goiás Goiânia GO Brasil Hospital do Coração de Goiás, Goiânia, GO – Brasil; 18 Hospital Santa Izabel Cabo Frio RJ Brasil Hospital Santa Izabel, Cabo Frio, RJ – Brasil; 19 Universidade Federal de Goiás Goiânia GO Brasil Universidade Federal de Goiás, Goiânia, GO – Brasil

**Table:** 

Posicionamento sobre Hipertensão Arterial e Espiritualidade – 2021	
O relatório abaixo lista as declarações de interesse conforme relatadas à SBC pelos especialistas durante o período de desenvolvimento deste posicionamento, 2020/2021.	
Especialista	Tipo de relacionamento com a indústria
Álvaro Avezum Jr.	Financiamento de atividades de educação médica continuada, incluindo viagens, hospedagens e inscrições para congressos e cursos, provenientes da indústria farmacêutica, de órteses, próteses, equipamentos e implantes, brasileiras ou estrangeiras:
	- EMS: Simpósios SBC Espiritualidade - DEMCA.
Andréa Araujo Brandão	Declaração financeira
	A - Pagamento de qualquer espécie e desde que economicamente apreciáveis, feitos a (i) você, (ii) ao seu cônjuge/ companheiro ou a qualquer outro membro que resida com você, (iii) a qualquer pessoa jurídica em que qualquer destes seja controlador, sócio, acionista ou participante, de forma direta ou indireta, recebimento por palestras, aulas, atuação como proctor de treinamentos, remunerações, honorários pagos por participações em conselhos consultivos, de investigadores, ou outros comitês, etc. Provenientes da indústria farmacêutica, de órteses, próteses, equipamentos e implantes, brasileiras ou estrangeiras:
	- Servier, Libbs e Merck: Hipertensão Arterial.
Audes Diógenes Magalhães Feitosa	Financiamento de atividades de educação médica continuada, incluindo viagens, hospedagens e inscrições para congressos e cursos, provenientes da indústria farmacêutica, de órteses, próteses, equipamentos e implantes, brasileiras ou estrangeiras:
	- EMS, Servier, Sandoz, Merck, Medtronic, Omron.
Celso Amodeo	Nada a ser declarado
Eduardo Costa Duarte Barbosa	Declaração financeira
	A - Pagamento de qualquer espécie e desde que economicamente apreciáveis, feitos a (i) você, (ii) ao seu cônjuge/ companheiro ou a qualquer outro membro que resida com você, (iii) a qualquer pessoa jurídica em que qualquer destes seja controlador, sócio, acionista ou participante, de forma direta ou indireta, recebimento por palestras, aulas, atuação como proctor de treinamentos, remunerações, honorários pagos por participações em conselhos consultivos, de investigadores, ou outros comitês, etc. Provenientes da indústria farmacêutica, de órteses, próteses, equipamentos e implantes, brasileiras ou estrangeiras:
	- EMS, Servier: Anti-hipertensivos; Cardios: Equipamentos.
	Outros relacionamentos
	Financiamento de atividades de educação médica continuada, incluindo viagens, hospedagens e inscrições para congressos e cursos, provenientes da indústria farmacêutica, de órteses, próteses, equipamentos e implantes, brasileiras ou estrangeiras:
	- Servier: Anti-hipertensivos.
Emilio Hideyuki Moriguchi	Declaração financeira
	A - Pagamento de qualquer espécie e desde que economicamente apreciáveis, feitos a (i) você, (ii) ao seu cônjuge/ companheiro ou a qualquer outro membro que resida com você, (iii) a qualquer pessoa jurídica em que qualquer destes seja controlador, sócio, acionista ou participante, de forma direta ou indireta, recebimento por palestras, aulas, atuação como proctor de treinamentos, remunerações, honorários pagos por participações em conselhos consultivos, de investigadores, ou outros comitês, etc. Provenientes da indústria farmacêutica, de órteses, próteses, equipamentos e implantes, brasileiras ou estrangeiras:
	- Amgen, AstraZeneca, Biolab, Kowa, MSD, Daiichi-Sankyo, Sanofi: Cardiologia.
	B - Financiamento de pesquisas sob sua responsabilidade direta/pessoal (direcionado ao departamento ou instituição) provenientes da indústria farmacêutica, de órteses, próteses, equipamentos e implantes, brasileiras ou estrangeiras:
	- Amgen, MSD, Daiichi-Sankyo, Kowa: Cardiologia.
	Outros relacionamentos
	Financiamento de atividades de educação médica continuada, incluindo viagens, hospedagens e inscrições para congressos e cursos, provenientes da indústria farmacêutica, de órteses, próteses, equipamentos e implantes, brasileiras ou estrangeiras:
	- Amgen, Biolab, Kowa, MSD, Daiichi-Sankyo, Sanofi: Cardiologia.
Fernando Antônio Lucchese	Nada a ser declarado
Fernando Nobre	Declaração financeira
	A - Pagamento de qualquer espécie e desde que economicamente apreciáveis, feitos a (i) você, (ii) ao seu cônjuge/ companheiro ou a qualquer outro membro que resida com você, (iii) a qualquer pessoa jurídica em que qualquer destes seja controlador, sócio, acionista ou participante, de forma direta ou indireta, recebimento por palestras, aulas, atuação como proctor de treinamentos, remunerações, honorários pagos por participações em conselhos consultivos, de investigadores, ou outros comitês, etc. Provenientes da indústria farmacêutica, de órteses, próteses, equipamentos e implantes, brasileiras ou estrangeiras:
	- Libbs, Novartis, Servier, Baldacci.
	Outros relacionamentos
	Financiamento de atividades de educação médica continuada, incluindo viagens, hospedagens e inscrições para congressos e cursos, provenientes da indústria farmacêutica, de órteses, próteses, equipamentos e implantes, brasileiras ou estrangeiras:
	- Libbs, Cristália, Daichi Sankio, Novartis, Biolab, Servier, Baldacci.
Hermilo Borba Griz	Declaração financeira
	A - Pagamento de qualquer espécie e desde que economicamente apreciáveis, feitos a (i) você, (ii) ao seu cônjuge/ companheiro ou a qualquer outro membro que resida com você, (iii) a qualquer pessoa jurídica em que qualquer destes seja controlador, sócio, acionista ou participante, de forma direta ou indireta, recebimento por palestras, aulas, atuação como proctor de treinamentos, remunerações, honorários pagos por participações em conselhos consultivos, de investigadores, ou outros comitês, etc. Provenientes da indústria farmacêutica, de órteses, próteses, equipamentos e implantes, brasileiras ou estrangeiras:
	- Pfizer, Bayer: Anticoagulação.
José Carlos Nicolau	Declaração financeira
	A - Pagamento de qualquer espécie e desde que economicamente apreciáveis, feitos a (i) você, (ii) ao seu cônjuge/ companheiro ou a qualquer outro membro que resida com você, (iii) a qualquer pessoa jurídica em que qualquer destes seja controlador, sócio, acionista ou participante, de forma direta ou indireta, recebimento por palestras, aulas, atuação como proctor de treinamentos, remunerações, honorários pagos por participações em conselhos consultivos, de investigadores, ou outros comitês, etc. Provenientes da indústria farmacêutica, de órteses, próteses, equipamentos e implantes, brasileiras ou estrangeiras:
	- Amgen: Hipolipemiante; Bayer: Anticoagulante; Daiichi Sankyo: Antiplaquetário, anticoagulante;
	Novartis: Inibidor SRA, hipolipemiante; Sanofi: Anticoagulante, hipolipemiante, antiplaquetário;
	Servier: antiplaquetário.
	B - Financiamento de pesquisas sob sua responsabilidade direta/pessoal (direcionado ao departamento ou instituição) provenientes da indústria farmacêutica, de órteses, próteses, equipamentos e implantes, brasileiras ou estrangeiras:
	- Astrazeneca: Antiplaquetário, hipoglicemiante; Bayer: Anticoagulante; Esperion: Hipolipemante; CSLBehring: hipolipemiante; Dalcor: Aumento HDL; Jansen: Anticoagulante; Novartis: Inibidor SRA; Novo Nordisk: Hipoglicemiante; Sanofi: Anticoagulate, hipolipemiante; Vifor: Deficiência de Ferro.
	C - Financiamento de pesquisa (pessoal), cujas receitas tenham sido provenientes da indústria farmacêutica, de órteses, próteses, equipamentos e implantes, brasileiras ou estrangeiras:
	- Roche: Kits de laboratório para avaliação de agregabilidade plaquetária.
	Outros relacionamentos
	Financiamento de atividades de educação médica continuada, incluindo viagens, hospedagens e inscrições para congressos e cursos, provenientes da indústria farmacêutica, de órteses, próteses, equipamentos e implantes, brasileiras ou estrangeiras:
	- Sanofi: Anticoagulante, antiplaquetário, hipolipemiante; Bayer: Anticoagulante; Servier: antiplaquetário.
Lucélia Batista Neves Cunha Magalhães	Nada a ser declarado
Marco Antônio Mota-Gomes	Declaração financeira
	A - Pagamento de qualquer espécie e desde que economicamente apreciáveis, feitos a (i) você, (ii) ao seu cônjuge/ companheiro ou a qualquer outro membro que resida com você, (iii) a qualquer pessoa jurídica em que qualquer destes seja controlador, sócio, acionista ou participante, de forma direta ou indireta, recebimento por palestras, aulas, atuação como proctor de treinamentos, remunerações, honorários pagos por participações em conselhos consultivos, de investigadores, ou outros comitês, etc. Provenientes da indústria farmacêutica, de órteses, próteses, equipamentos e implantes, brasileiras ou estrangeiras:
	- Omron, Beliva, Libbs.
	Outros relacionamentos
	Financiamento de atividades de educação médica continuada, incluindo viagens, hospedagens e inscrições para congressos e cursos, provenientes da indústria farmacêutica, de órteses, próteses, equipamentos e implantes, brasileiras ou estrangeiras:
	- Omron, Libbs.
Mario Henrique Elesbão de Borba	Nada a ser declarado
Mauro Ricardo Nunes Pontes	Declaração financeira
	A - Pagamento de qualquer espécie e desde que economicamente apreciáveis, feitos a (i) você, (ii) ao seu cônjuge/ companheiro ou a qualquer outro membro que resida com você, (iii) a qualquer pessoa jurídica em que qualquer destes seja controlador, sócio, acionista ou participante, de forma direta ou indireta, recebimento por palestras, aulas, atuação como proctor de treinamentos, remunerações, honorários pagos por participações em conselhos consultivos, de investigadores, ou outros comitês, etc. Provenientes da indústria farmacêutica, de órteses, próteses, equipamentos e implantes, brasileiras ou estrangeiras:
	- Novo Nordisk: Ozempic; Mantecorp: NesinaPio.
	Outros relacionamentos
	Financiamento de atividades de educação médica continuada, incluindo viagens, hospedagens e inscrições para congressos e cursos, provenientes da indústria farmacêutica, de órteses, próteses, equipamentos e implantes, brasileiras ou estrangeiras:
	- Servier: Acertanlo.
Paulo César Brandão Veiga Jardim	Nada a ser declarado
Pedro Pimenta de Mello Spineti	Declaração financeira
	B - Financiamento de pesquisas sob sua responsabilidade direta/pessoal (direcionado ao departamento ou instituição) provenientes da indústria farmacêutica, de órteses, próteses, equipamentos e implantes, brasileiras ou estrangeiras:
	- Boehringer Ingelheim: Empaglifozina.
	Outros relacionamentos
	Financiamento de atividades de educação médica continuada, incluindo viagens, hospedagens e inscrições para congressos e cursos, provenientes da indústria farmacêutica, de órteses, próteses, equipamentos e implantes, brasileiras ou estrangeiras:
	- Servier: Insuficiência cardíaca; Novartis: Entresto.
Ricardo Mourilhe-Rocha	Nada a ser declarado
Roberto Dischinger Miranda	Financiamento de atividades de educação médica continuada, incluindo viagens, hospedagens e inscrições para congressos e cursos, provenientes da indústria farmacêutica, de órteses, próteses, equipamentos e implantes, brasileiras ou estrangeiras:
	- EMS, Boehringher, Sanofi, Servier.
Roberto Esporcatte	Nada a ser declarado
Sérgio Lívio Menezes Couceiro	Declaração financeira
	A - Pagamento de qualquer espécie e desde que economicamente apreciáveis, feitos a (i) você, (ii) ao seu cônjuge/ companheiro ou a qualquer outro membro que resida com você, (iii) a qualquer pessoa jurídica em que qualquer destes seja controlador, sócio, acionista ou participante, de forma direta ou indireta, recebimento por palestras, aulas, atuação como proctor de treinamentos, remunerações, honorários pagos por participações em conselhos consultivos, de investigadores, ou outros comitês, etc. Provenientes da indústria farmacêutica, de órteses, próteses, equipamentos e implantes, brasileiras ou estrangeiras:
	- Pfizer, AstraZeneca, Servier, Medley: Palestras.
	Outros relacionamentos
	Financiamento de atividades de educação médica continuada, incluindo viagens, hospedagens e inscrições para congressos e cursos, provenientes da indústria farmacêutica, de órteses, próteses, equipamentos e implantes, brasileiras ou estrangeiras:
	- Aché, Pfizer, Servier, AstraZeneca: Palestras.
Weimar Kunz Sebba Barroso	Declaração financeira
	A - Pagamento de qualquer espécie e desde que economicamente apreciáveis, feitos a (i) você, (ii) ao seu cônjuge/ companheiro ou a qualquer outro membro que resida com você, (iii) a qualquer pessoa jurídica em que qualquer destes seja controlador, sócio, acionista ou participante, de forma direta ou indireta, recebimento por palestras, aulas, atuação como proctor de treinamentos, remunerações, honorários pagos por participações em conselhos consultivos, de investigadores, ou outros comitês, etc. Provenientes da indústria farmacêutica, de órteses, próteses, equipamentos e implantes, brasileiras ou estrangeiras:
	- EMS, Servier, Brace Pharma, Omron, Cardios: Hipertensão Arterial.
	B - Financiamento de pesquisas sob sua responsabilidade direta/pessoal (direcionado ao departamento ou instituição) provenientes da indústria farmacêutica, de órteses, próteses, equipamentos e implantes, brasileiras ou estrangeiras:
	- Ministério da Saúde, PROADI SUS: Hipertensão Arterial; Einstein: Projetos de Pesquisa.
	Outros relacionamentos
	Participação societária de qualquer natureza e qualquer valor economicamente apreciável de empresas na área de saúde, de ensino ou em empresas concorrentes ou fornecedoras da SBC:
	- Beliva Health.

## Conceitos Básicos sobre Espiritualidade e suas Formas de Avaliação

As definições de espiritualidade e religiosidade apresentam variações com o meio cultural, ambiental e religioso.^1^ A diversidade de conceitos é reconhecida, sem uma clara definição, sendo o termo usado de forma imprecisa e inconsistente com consequente dificuldade de medida.^2^

Para o Departamento de Estudos em Espiritualidade e Medicina Cardiovascular (DEMCA) da Sociedade Brasileira de Cardiologia (SBC), “Espiritualidade é um conjunto de valores morais, mentais e emocionais que norteiam pensamentos, comportamentos e atitudes nas circunstâncias da vida de relacionamento intra e interpessoais, e com o aspecto de ser motivado pela vontade e passível de observação e de mensuração”.^3^ O aspecto de mensuração da espiritualidade deve ser valorizado e aplicável a todos os indivíduos, independentemente de afiliação religiosa, incluindo ateus, agnósticos e aqueles com afiliação religiosa, mesmo sem a observância dos preceitos.^4^

Religiosidade é o quanto um indivíduo acredita, segue e pratica uma religião. Pode ser organizacional (participação na igreja, templo ou serviços religiosos) ou não organizacional, tais como rezar, ler livros ou assistir programas religiosos por iniciativa própria.^3^ Para Koenig, religião é “um sistema organizado de crenças, práticas e símbolos destinados a facilitar a proximidade com o transcendente ou o Divino, e fomentar a compreensão do relacionamento e das responsabilidades de uma pessoa com os outros que vivem em comunidade”.^1^^,^^5^ A religião é uma construção multidimensional que inclui crenças, comportamentos, dogmas, rituais e cerimônias que podem ser realizados ou praticados privada ou publicamente, de alguma forma derivados de tradições estabelecidas desenvolvidas dentro de uma comunidade.

O enfrentamento, ou *coping* religioso, é componente importante para entender o mecanismo pelo qual espiritualidade e religiosidade podem afetar a saúde: é o uso da religiosidade para se adaptar a desafios físicos, psicológicos e sociais em doenças e alterações da saúde.

## Escalas para Avaliação de Espiritualidade e Religiosidade

A espiritualidade/religiosidade pode ser avaliada na “anamnese espiritual”, que é o conjunto de perguntas feitas ao paciente para que compartilhe seus valores espirituais e religiosos, identificando possíveis questões espirituais que possam interferir na terapêutica. É necessário sempre centrar no paciente e guiar-se pelo que ele manifestar.^6^ Este componente da história clínica deve ser avaliado em todos os pacientes que procuram atendimento médico; a coleta da anamnese espiritual deve também ser realizada em todos os pacientes internados e/ou ambulatoriais, especialmente em doenças crônicas.

Muitos pacientes são religiosos ou espiritualizados e suas crenças os ajudam a lidar com a doença e a enfrentar situações adversas da vida, mas algumas situações trazem pontos de conflito com a prática médica. Nos períodos de hospitalização ou doença crônica, é frequente que fiquem afastados das comunidades e impedidos de praticar sua religião. Além disso, as crenças pessoais podem afetar decisões ligadas à saúde, por vezes alterando aspectos fundamentais do tratamento.^7^^,^^8^

Estudos demonstram que a maioria dos pacientes gostaria que seus médicos perguntassem sobre espiritualidade/religiosidade e, com isso, haveria mais empatia e confiança nos profissionais,^9^ levando a um cuidado mais humanizado^10^ e, consequentemente, mais participativo de ambas as partes quando avaliado como fator social e demográfico.^7^^,^^8^

O objetivo é entender as crenças, identificar aspectos que podem interferir nos cuidados de saúde do paciente, avaliar a força espiritual individual/social/familiar que lhe permitirá enfrentar a doença, oferecer empatia e suporte, ajudando-o a encontrar aceitação da doença, e, ainda, identificar situações de conflito ou sofrimento que exigirão avaliação por profissional treinado.^11^^,^^12^ Existem várias maneiras de abordar o tema – o mais importante é fazer de forma sensível, sem jamais impor ou promover a religião e nem tampouco prescrever orações ou práticas religiosas, coagindo o paciente a adotar crenças ou práticas específicas. Religião não deve ser prescrita, forçada ou encorajada, para não acrescentar culpa a um possível fardo provocado pela doença. O bom senso deve ser usado como regra. Nas situações extremas, como acidentes graves ou infarto agudo do miocárdio (IAM), pode levar ao estresse e até mesmo piorar a evolução do paciente.^13^^,^^14^

Não havendo adequado preparo do médico ou não aceitação por parte do paciente com relação a esse tipo de abordagem, é natural que não deva ser feita. Para indivíduos não religiosos ou adversos ao tema, deve-se entender como enfrentam e convivem com a doença, o que promove um propósito e significado para suas vidas (família, amigos, *hobby* etc.) e quais crenças culturais podem ter impacto sobre seus tratamentos.^15^

### Escalas e Instrumentos

Os vários instrumentos psicométricos podem ser divididos em diferentes categorias:^16^^,^^17^

#### Rastreamento Espiritual

Observa a presença de necessidades espirituais que indiquem uma avaliação mais profunda. É breve e de fácil aplicação (Quadro 1).

**Quadro 1:** Instrumentos de rastreamento espiritual

Ferramentas de rastreamento	Domínios espirituais avaliados
Protocolo “Rush” de rastreamento espiritualidade/religiosidade^17^	Importância da espiritualidade/ religiosidade na doença; força ou conforto espiritual
“Você está em paz?”^18^	Paz interior
“Você sente dor ou sofrimento espiritual?”^19^	Dor/sofrimento espiritual
Escala de injúria espiritual^20^	Culpa, raiva, tristeza, sentimento de injustiça, medo da morte

#### Instrumentos para Coleta da História Espiritual

Permitem avaliação mais ampla dos diferentes domínios da espiritualidade/religiosidade que poderão afetar evolução clínica, postura, autocuidado e bem-estar físico, mental e espiritual perante a doença. São instrumentos bem estruturados, abordam diferentes domínios, mas devem ser aplicados de memória, de modo informal, ao longo da conversa. Servem como ferramenta ou guia e não devem ser vistos com rigidez, mas como aprendizado contínuo e consequente familiarização com a tarefa de completar a anamnese. Existem vários instrumentos validados para coleta, seja com objetivo de avaliar espiritualidade ou religiosidade ou para pesquisa.

#### Escalas de Religiosidade

O Índice de Religiosidade da Universidade de Duke (DUREL)^21^ é uma escala com cinco itens que mensura três dimensões do envolvimento religioso: O primeiro item (1) avalia religiosidade organizacional; o segundo (2) avalia religiosidade não organizacional; e os itens 3, 4 e 5 contemplam a avaliação da religiosidade intrínseca. O DUREL é sucinto e de fácil aplicação, validado no Brasil^22^ e aborda os principais domínios da religiosidade. As dimensões de religiosidade mensuradas pelo DUREL têm se mostrado relacionadas a diversos indicadores de suporte social e saúde.^22^

#### Ferramentas para Avaliar História Espiritual

Envolvem um conjunto de questões sobre diversos domínios que se associam a desfechos de saúde, baseadas em escalas previamente validadas. As principais escalas são: FICA,^23^ FAITH,^24^ SPIRIT^25^ e HOPE.^26^ O questionário FICA, criado por médicos, é o que tem demonstrado melhor característica psicométrica, pode ser usado em diferentes situações, avalia quatro dimensões (fé ou crenças, importância e influência, comunidade e ação no tratamento) e é de fácil aplicação, de rápida execução e memorização.^27^

Espera-se que, após a anamnese espiritual, face as informações obtidas, o profissional sinta-se capacitado a orientar o paciente, agregando os valores e recursos de espiritualidade/religiosidade na condução clínica.

Sempre com total respeito às crenças do paciente e sem proselitismo, abordagens de questões religiosas podem se mostrar complexas, com potencial conflito, e devem ser recebidas com empatia e compreensão. Nessas circunstâncias, deve-se considerar a contribuição advinda da visita do líder religioso. Identificadas ferramentas provindas da espiritualidade, os pacientes devem ser estimulados a utilizá-las como forma de prevenção das doenças. Tais práticas frequentemente não se restringem a orações e meditação, mas também leitura, música etc. No mesmo sentido, deve-se ajudar o paciente a identificar aspectos espirituais que, juntamente com o tratamento padrão, possam auxiliar no desfecho da doença. No caso de doenças graves o médico pode ajudar a encontrar significado e aceitação, e enfrentar a situação usando os seus recursos espirituais da melhor forma. Muitas vezes, de tais interações, surgem alternativas e adequações do plano terapêutico, em que se fortalecem os sentidos de autonomia e cuidados centrados no paciente.^27^^,^^28^

**Mensagens principais**

**Table:** 

**Conceitos**
**Espiritualidade** é um conjunto de valores morais, mentais e emocionais que norteiam pensamentos, comportamentos e atitudes nas circunstâncias da vida de relacionamento intra e interpessoais, e com o aspecto de ser motivado pela vontade e passível de observação e de mensuração.
**Religiosidade** é quanto um indivíduo acredita, segue e pratica uma religião.
**Aplicação prática**
Havendo disposição e capacidade do profissional e do paciente, a avaliação de espiritualidade e religiosidade deve ser sempre buscada.
A avaliação deve ser realizada por meio de questionários e escalas que devem ser de domínio do profissional de saúde para aplicação.
**Como proceder após a obtenção da anamnese espiritual**
Com as informações da dimensão espiritual, é possível ampliar o entendimento do impacto da espiritualidade/religiosidade no curso da doença e identificar demandas dos pacientes nesta área.

## Mecanismos Envolvendo a Espiritualidade e a Fisiopatologia da Hipertensão Arterial

A hipertensão arterial (HA) é caracterizada como uma doença multifatorial e multigênica que compromete principalmente o sistema vascular, das pequenas e das grandes artérias, levando a danos progressivos e, após certo ponto, irreversíveis aos chamados órgãos-alvo (coração, rins e cérebro).^29^ Mecanismos biológicos neurais, hormonais, renais e vasculares participam e interagem de diferentes maneiras para resultar na elevação sustentada da pressão arterial (PA) em cada indivíduo. Múltiplos fatores estão envolvidos e influenciam os sistemas biológicos, tais como: genéticos, ambientais, humorais, hemodinâmicos, neurais, endócrinos, anatômicos, adaptativos e, mais recentemente, a inflamação e a produção de espécies reativas de oxigênio têm sido implicadas como base para a atuação de vários desses fatores em nível celular e molecular.^30^

Os mecanismos envolvidos no controle da PA dependem de estímulos intrínsecos e extrínsecos. Sabe-se que, diante de situações de estresse, ocorre ativação do sistema nervoso simpático com maior descarga de substâncias vasoativas que vão facilitar o aparecimento de HA.^30^ Em situações de estresse e ansiedade, associam-se erros alimentares, com ingestão excessiva de carboidratos, gordura e sal, o que também contribui para elevação da PA. Assim, é de grande importância a compreensão do humano como um ser biopsicossocial, dimensões que se inter-relacionam fortemente. Admite-se que a espiritualidade tem influência sobre essa tríade e participa desse equilíbrio multifatorial.^3^

Ativação do sistema nervoso simpático (SNS) a longo prazo, do sistema renina angiotensina aldosterona, mecanismos renais alterados, retenção de sódio e alterações vasculares representadas pela disfunção endotelial e alterações da camada muscular lisa vascular se inter-relacionam e estão fortemente implicados no desenvolvimento e na manutenção da HA pelo aumento sustentado da resistência vascular periférica, aumento dos níveis de marcadores inflamatórios e lesões em órgãos-alvo.^30^

Estudos têm investigado a relação de várias dimensões de espiritualidade/religiosidade com a PA, e a maioria mostra associação com menores valores de PA e menor taxa de HA,^1^^,^^31^ com maior efeito sobre a redução da PA diastólica (PAD).^4^ É possível que indivíduos mais conectados com espiritualidade/religiosidade tenham sentimentos mais positivos (amor, paz, perdão) que negativos (medo, ansiedade, depressão) e este aspecto promova resposta de redução da atividade do SNS e dos níveis de cortisol. No entanto, os estudos não são totalmente conclusivos.^1^^,^^31^^,^^32^

Práticas de espiritualidade/religiosidade no enfrentamento do estresse associaram-se a menor incidência de HA, especialmente em presença de níveis maiores de estresse^33^ e redução dos níveis de proteína C reativa (PCR) em populações afro-americanas.^34^

Metanálise envolvendo 87 estudos investigou o impacto de práticas de espiritualidade/religiosidade sobre marcadores fisiológicos de saúde e mostrou que havia associação inversa entre medidas de espiritualidade/religiosidade, em especial a participação em cultos, religiosidade intrínseca, preces e meditação com menor estresse, PA e marcadores inflamatórios (PCR), embora o tamanho do efeito fosse pequeno e com grande heterogeneidade interestudos, sugerindo a presença de fatores de confusão.^35^

**Mensagens principais**

**Table:** 

**Conceitos**
Estudos clínicos que investigaram a relação de espiritualidade/religiosidade com a pressão arterial e marcadores biológicos são observacionais e a maioria sugere menores valores de pressão arterial, menores níveis de marcadores inflamatórios, redução da atividade simpática e dos níveis de cortisol.
**Aspectos práticos**
Práticas de espiritualidade/religiosidade, em especial a participação em cultos, religiosidade intrínseca, preces e meditação associaram-se inversamente com marcadores fisiológicos de saúde, tais como menos estresse, menores valores de pressão arterial e de marcadores inflamatórios (PCR).

## Associação entre Espiritualidade, Hipertensão Arterial e Evidências em Prevenção Primária

Estudos que avaliaram a influência de fatores psicológicos e relacionados à espiritualidade/religiosidade sobre o aumento da PA têm buscado adicionar conhecimento sobre a participação desses fatores na incidência e na prevalência de HA, porém os resultados não são concordantes.^36^

A mensuração objetiva de sentimentos como propensão ao perdão, otimismo, pessimismo, hostilidade, empatia, estados de paz e traços de estresse emocional, envolvidos na espiritualidade, é difícil. Muitas vezes, o grau de espiritualidade é quantificado pela frequência a atos e compromissos com a comunidade religiosa ou tempo de leitura relacionados aos temas da religião de escolha. Assim, apresentaremos em muitos momentos evidências de espiritualidade/religiosidade de forma indistinta, porque os estudos assim o fizeram.

No Chicago Community Adult Health Study, constatou-se que maior presença de indicadores de religiosidade não estava associada à proteção para o desenvolvimento de HA. Entretanto, foram encontrados valores mais baixos de PAD nos indivíduos compropensão ao perdão, comparados aos não propensos a esse sentimento.^37^

Nesta mesma direção, o estudo SWAN (Study of Women’s Health Across the Nation), envolvendo 1.658 mulheres, utilizou a escala de experiências espirituais (Daily Spiritual Experiences Scale) e não mostrou diferença estatística entre as experiências diárias de espiritualidade e a prevalência/incidência de HA em 3 anos.^38^

Em outro estudo de coorte longitudinal de base populacional de idosos, Koenig e col. encontraram forte relação transversal entre envolvimento religioso e PA mais baixa; no entanto, no seguimento longitudinal, a redução da PA não foi significativa.^39^

Por outro lado, o Black Women’s Health Study, grande estudo de coorte com acompanhamento de 59.000 mulheres afrodescendentes por 23 anos, demonstrou que o intenso envolvimento da espiritualidade/religiosidade no enfrentamento de eventos estressantes em comparação com nenhum envolvimento foi associado a menor risco de desenvolver HA. A associação foi mais forte entre as mulheres que relataram maiores níveis de estresse percebido. Por sua vez, oração frequente foi associada a aumento do risco de HA.^33^

Estudo realizado no Brasil, envolvendo comunidade com alta religiosidade, evidenciou menor prevalência de HA nesses indivíduos que a prevalência nacional.^40^ Em estudo utlizando monitoramento ambulatorial da PA (MAPA), em uma coorte de 100 participantes, Holt-Lunstad e col. demonstraram que um alto nivel de espiritualidade associou-se a menores valores de PA.^41^

No inquérito NHANES III, 14.475 homens e mulheres americanas com 20 anos de idade ou mais tiveram a PA avaliada e relataram frequência de comparecimento a serviços religiosos e histórico de tratamento da HA. Em comparação aos que nunca participaram de serviços religiosos, os participantes que frequentavam semanalmente essas atividades tiveram PA sistólica (PAS) 1,46 mmHg (IC 95%, 2,33, 0,58 mmHg, p < 0,01) menor, enquanto as pessoas que compareciam em atividades do gênero mais que 52 vezes/ano tinham PAS 3,03 mmHg (IC 95%, 4,34, 1,72 mmHg, p < 0,01) mais baixa. Nenhuma modificação significativa do efeito por gênero foi observada; essas estimativas são ajustadas para uma interação significativa entre a idade e a frequência menor que semanal (1 a 51 vezes) (p < 0,05).^42^

As relações entre raça/etnia e HA, bem como frequência de atendimento a serviços religiosos e HA, não estão plenamente esclarecidas. Esta inter-relação foi avaliada em brancos e negros não hispânicos e hispânicos (N = 12.488). Em comparação com aqueles que nunca compareceram aos serviços religiosos, os brancos que compareceram semanalmente apresentaram menor chance de HA, assim como os negros que compareceram mais de uma vez por semana. Não havia relação entre frequência a serviços religiosos e HA entre hispânicos, sugerindo que esses benefícios não são semelhantes para todos.^43^

Um estudo transversal recrutou 1.384 adultos budistas tibetanos de dois institutos budistas na província de Sichuan na China, e incluiu 798 residentes tibetanos adultos de vilas e cidades próximas. O risco de HA nos budistas foi significativamente menor, em 38%, em comparação com os não budistas. Maior tempo de participação nas atividades budistas mostrou-se associado à diminuição da prevalência de HA, bem como menor PA em análise de um subgrupo de 570 budistas.^44^ Em estudo longitudinal, Timio e col. compararam a PA de freiras reclusas de ordem secular com mulheres leigas. Os dados basais foram semelhantes para todas as variáveis, mas, ao fim de 30 anos, observaram-se aumentos significativos de PAS e PAD no segundo grupo.^45^ Portanto, esses estudos sugerem o papel protetor da espiritualidade/religiosidade na HA, mediado por comportamentos que poderiam ser aplicados à população em geral.^44^^,^^45^

Em estudo que examinou a hostilidade e o risco de doença cardiovascular (DCV) em jovens hispânicos, Sethness e col. encontraram que esses sentimentos ruins e agressivos associaram-se com PA mais elevada.^46^

No estudo Biopsychosocial Religion and Health Study (N = 9.581), fatores de estilo de vida, como dieta vegetariana e exercícios regulares, foram importantes preditores de taxas reduzidas de HA; a religiosidade intrínseca, mesmo após controle desses fatores, esteve tão fortemente relacionada às menores taxas de HA quanto esses fatores. Esse estudo demonstra que, além dos efeitos positivos das escolhas de estilo de vida sobre a saúde observados no grupo, a religião pode oferecer efeitos salutares diretos sobre a HA.^47^

O ensaio prospectivo Nurses’ Health Study II avaliou 44.281 mulheres não hipertensas, observando-se que a participação em serviços religiosos foi, de forma modesta, inversamente associada à HA com efeito dose-resposta, ou seja, quanto maior a frequência, menor a incidência de HA. A frequência por mais de uma vez por semana, comparada a nenhuma ou quase nenhuma participação, apresentou taxa de risco de 0,91 (IC 95%, 0,86, 0,97).^48^

**Mensagens principais**

**Table:** 

**Conceitos**
Estudos que avaliaram a influência de fatores psicológicos e relacionados à espiritualidade/religiosidade sobre o aumento da pressão arterial têm buscado adicionar conhecimento a respeito da participação desses fatores na hipertensão arterial, mas os resultados não são concordantes No ensaio prospectivo Nurses’ Health Study II, avaliando 44.281 mulheres não hipertensas, as mulheres que frequentavam serviços religiosos eram menos propensas a desenvolver hipertensão arterial.
**Aspectos práticos**
Embora os resultados de estudos avaliando espiritualidade/religiosidade não sejam todos concordantes quanto a menor ocorrência de hipertensão arterial em pessoas que têm prática de espiritualidade/religiosidade, alguns deles demonstraram menores valores de pressão arterial sistólica e menor incidência de hipertensão arterial, diretamente relacionados à frequência de atendimento a práticas religiosas.

## Hipertensão Arterial, Espiritualidade e Outros Determinantes Psicossociais

Fatores psicossociais devem ser colocados como parte do perfil de risco do indivíduo. Para estabelecer a relação de fatores psicossociais com a ocorrência de HA, podemos acompanhar grandes amostras prospectivamente com medidas-padrão de PA e variáveis psicossociais.^49^ Esses estudos epidemiológicos fornecem evidências sobre a influência psicossocial, mas não fornecem dados sobre mecanismos fisiopatológicos. Diferenças na magnitude ou padrão de reações a estímulos comportamentais podem ser relacionadas ao risco de desenvolver HA.

Estudos experimentais permitem avaliar o impacto dos estímulos psicológicos a serem examinados sob condições cuidadosamente controladas. Contudo, há limitações importantes, pois as respostas podem ser apenas agudas. O terceiro tipo de investigação é avaliar a medida da PA no cotidiano, relacionando as condições psicossociais e verificando o impacto delas. A dificuldade deste método é que a PA no cotidiano é afetada por uma ampla gama de fatores, tais como exercício, alimentação e outros hábitos de vida. O modelo deve sofrer um tratamento estatístico adequado para distinguir a influência de fatores psicossociais.^49^ Há estudos com pontos fortes, mas, também, com limitações. Para termos uma avaliação da influência dos fatores psicossociais, é necessário observar e integrar as diversas abordagens.

### Características Psicossociais

Uma série de características psicológicas tem sido associada à HA, incluindo ansiedade, raiva, hostilidade, saúde mental, problemas sociais, no trabalho e em casos de depressão.^49^^,^^50^ Contudo, a evidência mais consistente é para características relacionadas com raiva e hostilidade.^49^

Traços relacionados com a raiva podem não ser aparentes o tempo todo, mas revelam-se quando as pessoas são confrontadas ou em momentos nos quais se sentem ameaçados. A raiva é considerada relevante para o desenvolvimento de HA ou mesmo em indivíduos que experimentam estresse crônico causado por suas condições de vida ou fatores de trabalho. No estudo de Casagrande e col., foi demonstrado, utilizando a MAPA, que sentimentos sustentados de raiva concorreram para ausência de descenso da PA durante o sono, que é relacionado a pior prognóstico.^51^

Em outro estudo, jovens e adultos submetidos a tarefas desafiadoras foram avaliados antes e após a tarefa.^52^ Os Indivíduos com alta ou baixa inibição da raiva não apresentavam diferença em condições de repouso. No entanto, em resposta à tarefa comportamental, aqueles que inibiam a raiva eram mais reativos. Interessante que este foi o padrão entre os participantes com histórico familiar de HA, sugerindo que fatores genéticos também interagem com disposições psicológicas e situacionais que podem influenciar o risco de desenvolver HA.

### Características do Trabalho

Estudos demonstraram que certos tipos de trabalho estão associados ao risco de desenvolver HA. Os indivíduos de maior risco são aqueles cujos empregos têm demandas que superam as recompensas.^49^ Estudo de caso-controle envolvendo homens de 30 a 60 anos de idade trabalhando em diferentes locais em Nova York mostrou que a tensão no trabalho foi um determinante independente de HA após controle de idade, índice de massa corporal, comportamento psicológico do tipo A, excreção de sódio por 24 horas, atividade física no trabalho, educação, tabagismo e consumo de álcool.^53^ Após 3 anos, aqueles com alto nível de estresse no emprego persistentemente apresentavam maior PA. Essa resposta pode ter relação com o ritmo que é imposto à realização da tarefa e a sua relação com supressão do barorreflexo quando o grau de exigência é mínimo.^49^

A insegurança no emprego pode gerar raiva e indisposições psicológicas como aquela oriunda por trabalhar em empresas com altas taxas de demissão, gerando apreensão entre os funcionários.^50^ Interessante que a carga de trabalho não parece ter relação com PA mais alta.^50^

O estudo CARDIA, que avaliou 8.395 trabalhadores de “colarinho branco” no Canadá, observou tanto a exposição cumulativa quanto a nova carga de trabalho. Aumentos na PA, após 7,5 anos de acompanhamento, foram observados entre os trabalhadores com baixos níveis de apoio social no trabalho.^54^ Também foi observado em estudo observacional que um aumento de salário considerável pode reduzir em 16% o risco de desenvolver HA.^50^

### Isolamento e Suporte Social

O apoio social é um processo importante, por meio do qual lidamos com a vida e suas adversidades, e o isolamento, definido em termos de tamanho e composição da rede social (p. ex., estado civil, número de amigos e parentes próximos, religiosos ou outros grupos) tem sido associado a doenças e mortalidade cardiovasculares.^49^ O isolamento ou baixo apoio social está associado à PA mais alta.^49^ As relações sociais são importantes fontes de apoio emocional e prático, e podem amortecer os efeitos psicológicos do estresse. A falta de apoio e de relacionamentos não apenas deixam a pessoa sem esses recursos, mas pode ser uma importante fonte de estresse.

Dos vários elementos da rede social, o casamento é frequentemente o relacionamento central da vida das pessoas. Os indivíduos casados tendem a ter melhores resultados nas avaliações de saúde que aqueles que vivem sós, e outras fontes de apoio não compensam totalmente os efeitos da solidão. Por outro lado, os relacionamentos podem ser uma fonte de conflito, e o estresse associado a casamentos infelizes ou tensos tem sido associado a efeitos cardiovasculares (CV) danosos. Os episódios agudos de conflito conjugal têm demonstrado produzir elevações da PA.^54^

### Saúde Mental

Pacientes que reexperimentam sintomas relacionados a um trauma prévio apresentam taxas significativamente mais altas de HA.^50^ Um estudo mostra que o estresse percebido pode ser considerado um fator de risco para HA em mulheres com ocupação de baixa renda. Estudos que abordam as relações entre estresse e HA devem salientar possíveis interações com gênero e *status* ocupacional.^55^

Experiências associadas ao racismo podem afetar adversamente a saúde, tendo sido demonstrado que pode haver aumento da incidência de HA associado a experiências de racismo em certos subgrupos de mulheres afro-americanas.^56^

O estresse agudo promove elevação transitória da PA, mas não há evidências consistentes de que esse efeito resulte em HA sustentada. Estresse crônico e, particularmente, a resposta não adaptativa ao estresse são causas mais prováveis de desenvolver HA.^57^ Uma metanálise de estudos de coorte avaliou o efeito do estresse emocional na elevação da PA, tendo demonstrado que indivíduos que tiveram respostas mais intensas às tarefas estressoras eram 21% mais propensos a desenvolver aumento da PA (OR: 1,21; IC 95%, 1,14-1,28; p < 0,001). Embora a magnitude do efeito tenha sido pequena, os resultados sugerem a relevância no controle do estresse psicológico para não ocorrer elevação da PA.^58^

### Depressão e Distúrbios do Sono

Existem resultados inconsistentes na literatura quanto à associação de depressão com HA. Dois estudos avaliaram possível relação entre depressão e risco de desenvolver Hipertensão Arterial. No estudo de coorte Whitehall, os participantes do grupo de “depressão crescente” (caracterizado por aumento do número de episódios depressivos ao longo dos anos) tiveram um risco 25% menor de HA na idade de 35 a 39 anos quando comparados aos do grupo de “depressão baixa/transitória”. No entanto, houve aumento mais rápido de HA relacionado à idade no grupo de “depressão crescente”, correspondendo a um aumento 7% maior na chance de HA a cada aumento de 5 anos na idade. Assim, o risco de HA em participantes do grupo de “depressão crescente” no final do acompanhamento foi substancialmente maior do que no grupo de “depressão baixa/transitória”. O padrão foi mais significativo nos homens do que nas mulheres. Este estudo sugere que o risco de HA aumenta com a experiência repetida de episódios depressivos ao longo do tempo e se torna evidente na idade adulta mais avançada.^59^

O NHANES I 1982-1992^60^ examinou os efeitos da duração do sono e insônia na associação entre depressão e HA; mas, como no estudo anterior, os resultados dependiam da idade da coorte. Indivíduos com 32 a 59 anos de idade com depressão apresentaram 44% maior risco de terem HA nos 10 anos de acompanhamento (RC: 1,44, IC: 1,15-1,80). Indivíduos que relataram dormir 5 horas ou menos por noite tiveram 50% mais chances de ter HA quando comparados com aqueles que dormiam de 7 a 8 horas por noite (RC: 1,50, IC: 1,11-2,02). Não houve associação entre depressão, qualidade do sono e incidência de HA em idosos (idades de 60 a 86 anos).

Todos os estudos mostram uma relação direta entre os distúrbios do sono e incidência de HA. No Penn State Cohort, o risco de incidência de HA em pessoas com curta duração ou distúrbios do sono foi significativamente maior, mas tornou-se marginalmente significativo após o controle da obesidade (OR: 1,6, IC 95%, 0,9-2,8). Insônia crônica está associada a um risco aumentado da incidência de HA. Assim, é possível que a duração do sono possa ser considerada um determinante de HA.^61^

### Personalidade

A personalidade e o risco de desenvolver HA também apresentam resultados controversos. A personalidade do tipo D, caracterizada por altos níveis de afetividade negativa e alta inibição, não se associou a pressões arteriais sistólica e/ou diastólica elevadas. Entretanto, níveis mais baixos de consciência caracterizada por desorganização, irresponsabilidade, indisciplina, emoções negativas e reação exagerada ao estresse estiveram associados à HA.^50^

**Mensagens principais**

**Table:** 

**Conceitos**
São fatores psicossociais intervenientes no comportamento da PA e, por conseguinte, no aparecimento de HA: personalidade, tipo de trabalho, isolamento social, saúde mental, depressão e distúrbios do sono
**Aspectos práticos**
A avaliação e a abordagem dessas condições devem ser feitas no sentido de identificar quando podem interferir no comportamento da PA e no controle da HA.

## Espiritualidade na Adesão e no Tratamento de Hipertensão Arterial

Ao lado do tratamento não medicamentoso estão medidas que envolvem o uso de medicamentos anti-hipertensivos de reconhecida eficácia e efetividade para o tratamento da HA.^3^^,^^29^^,^^62^ O controle da PA modifica a história natural da doença, diminuindo sua morbidade e mortalidade. Em todo o mundo, esse objetivo encontra uma barreira, de difícil transposição, que é a falta de adesão às orientações de mudanças de estilo de vida ou prescrições de medicamentos.^3^^,^^29^^,^^62^ Entre os aspectos culturais que interferem nas atitudes dos indivíduos, estão aqueles reconhecidos como espiritualidade/religiosidade.^63^^–^^68^

Há mais de 4 décadas, inúmeros estudos demonstram que espiritualidade, no seu sentido mais amplo, perpassando aspectos da própria religiosidade, mas, principalmente, reforçando o sentimento de autoconhecimento, autoconfiança, resiliência, crença na possibilidade da autodeterminação, com uma visão positiva do mundo e do futuro, traz benefícios incontestes para melhor adesão às orientações dadas pelos profissionais de saúde.^63^^,^^66^^,^^69^^,^^70^

A espiritualidade tem forte influência sobre alguns hábitos de vida que, por sua vez, estão diretamente relacionados à HA. Diferentes práticas espiritualistas foram avaliadas em hipertensos, com influência nos hábitos e qualidade de vida.^31^^,^^71^ Diversos estudos clínicos têm demonstrado um efeito benéfico, por exemplo, da meditação transcendental (MT) na redução da PA em pacientes hipertensos.^72^^,^^73^ Uma metanálise desses estudos aleatorizados mostrou que a MT reduz a PA entre 4 e 6 mmHg, de uma forma dose-dependente (quanto mais sessões de MT, maior o efeito na redução da PA).^74^

Um importante estudo clínico aleatorizou 201 individuos negros com doença arterial coronariana (DAC) para um programa de MT ou educação em saúde. No seguimento médio de 5 anos, houve redução de risco de 48% na incidência do desfecho primário de morte, acidente vascular encefálico (AVE) IAM (RC: 0,52, IC 95%, 0,29-0,92, p = 0,025). Houve também redução da PA sistólica média (5 mmHg) e na tendência à raiva medida por escalas específicas (p < 0,05). A conclusão do estudo é que a MT pode ser benéfica no manejo da HAS e na prevenção secundária de DCV.^75^

Intervenções baseadas em ioga têm sido estudadas no manejo de DCV. Uma metanálise de 17 estudos mostrou que a ioga se associou à redução significativa das pressões arteriais sistólica e diastólica, especialmente se os três elementos da ioga forem usados (posições, respiração e meditação).^76^

Apesar de as evidências mostrarem ser menos robusto o impacto de intervenções baseadas em espiritualidade/religiosidade diretamente sobre a HA, já há uma significativa base de informações mostrando associação benéfica entre aspectos religiosos (afiliação religiosa, frequência a atividades religiosas organizadas ou privadas) ou espirituais (tendência a otimismo e gratidão, emoções positivas) e redução da incidência de HA, ou melhor controle da PA nos indivíduos já hipertensos.^77^^,^^78^

A coorte Black Women’s Health Study mostrou que as situações de espiritualidade/religiosidade contribuem com uma modulação mais suave de situações da vida cotidiana e trazem benefícios no controle da PA.^33^

As relações entre espiritualidade/religiosidade e adesão ao tratamento medicamentoso em doenças crônicas podem variar conforme as populações estudadas, mas, de forma geral, há influência positiva.^1^^,^^79^^–^^82^ A espiritualidade pode levar à melhora na adesão aos medicamentos pelo melhor controle de sentimentos negativos, como ansiedade, estresse e depressão.^65^

Em estudos qualitativos de pequeno porte, mulheres da comunidade negra acreditavam que a espiritualidade ajudava na adesão ao tratamento medicamentoso.^83^^,^^84^ Por outro lado, em outra população de mulheres negras, a espiritualidade/religiosidade esteve associada à pior adesão ao tratamento da HA não usando medicamentos, pela crença de uma cura divina.^85^

Em outros estudos, não houve correlação entre espiritualidade/religiosidade com a adesão nem com a confiança no profissional de saúde. Contudo, a confiança no médico estava associada a maior adesão aos medicamentos anti-hipertensivos.^67^^,^^86^ Outros fatores podem também influenciar na adesão ao tratamento proposto, tais como autoeficácia, crenças culturais e o grau de conhecimento sobre a doença.^87^

É fundamental que os profissionais de saúde compreendam a importância da espiritualidade/religiosidade, reconheçam e valorizem os aspectos culturais e respeitem as crenças e esperanças. Esta atitude, aparentemente simples, trará muitas e maiores possibilidades da obtenção de sucesso na empreitada do tratamento do paciente portador de doença crônica, pois essa realidade exige muito mais disciplina, perseverança e fé.

**Mensagens principais**

**Table:** 

**Conceitos**
Há uma significativa base de informações mostrando associação benéfica entre aspectos religiosos (afiliação religiosa, frequência a atividades religiosas organizadas ou privadas) ou espirituais (tendência a otimismo e gratidão, emoções positivas) e redução da incidência de hipertensão arterial ou melhor controle da pressão arterial.
Há mais de 4 décadas já se demonstrou que religiosidade, reforçando o sentimento de autoconhecimento, autoconfiança, resiliência, crença na possibilidade da autodeterminação, com uma visão positiva do mundo e do futuro, traz benefícios para melhor adesão às orientações dadas pelos profissionais de saúde.
**Aspectos práticos**
É fundamental que os profissionais de saúde compreendam a importância da espiritualidade/religiosidade, reconheçam e valorizem os aspectos culturais e respeitem as crenças e esperanças.
Esta atitude, aparentemente simples, trará muitas e maiores possibilidades da obtenção de sucesso no tratamento do paciente portador de doença crônica, pois essa realidade exige muito mais disciplina, perseverança e fé.

## Espiritualidade e Eventos Cardiovasculares Relevantes

A associação entre espiritualidade, religiosidade e desfechos clinicamente relevantes em diferentes áreas da saúde tem sido objeto de estudos observacionais desde os anos 1980;^88^ destaque especial tem sido dado às DCV.

O Estudo InterHeart avaliou fatores de risco associados independentemente com IAM em 52 países, incluindo o Brasil, e demonstrou que 90% do risco atribuível da população (RAP) era associado a nove fatores de risco simples de identificação e passíveis de modificação (dislipidemia, tabagismo, obesidade abdominal, diabetes, HA, sedentarismo, alimentação não saudável, álcool e fatores psicossociais). Dentre os fatores de risco, aqueles denominados psicossociais (estresse e depressão) foram responsáveis por 33% do RAP associado a IAM, impacto este maior do que fatores de risco consagrados como HA e diabetes.^89^ Complementarmente, o InterStroke avaliou os fatores de risco independentes associados com AVE no mundo. Seguindo a mesma estratégia do InterHeart, observou-se que os fatores psicossociais, estresse e depressão foram responsáveis por 17% do RAP associado ao AVE no mundo.^90^

No ano 2000, metanálise de 42 estudos, incluindo cerca de126.000 indivíduos, demonstrou que o envolvimento religioso estava associado a menor taxa de mortalidade por todas as causas.^91^ Estudos subsequentes demonstraram que existe redução de mortalidade com a frequência de participação em serviços religiosos, e que esta associação era substancialmente mediada por comportamentos de saúde e modificação de outros fatores de risco.^42^^,^^92^

Dois grandes estudos de coorte publicados em 2016 e 2017 reforçaram esses achados. No ensaio Black Women’s Health Study com 36.613 mulheres negras norte-americanas, observou-se redução de 46% na taxa de mortalidade, comparando-se a participação em serviços religiosos várias vezes por semana com nenhuma participação. Não foi observada associação entre frequência diária de orações, enfrentamento religioso ou autoidentificação como pessoa muito religiosa/espiritualizada com mortalidade.^93^

No ensaio Nurses’ Health Study, mais de 74.000 enfermeiras foram acompanhadas por até 8 anos, observando-se que a participação em serviços religiosos pelo menos uma vez por semana comparada com nenhuma frequência foi associada à redução da mortalidade por todas as causas, mortalidade por DCV e por câncer em cerca de 30%.^94^

Por outro lado, em coorte com 2.068 pacientes admitidos por síndrome coronariana aguda, não se observou associação entre conforto pela religião, orações pela própria saúde ou preces feitas por outros, e sobrevida em até 2 anos de seguimento.^95^ Os pacientes que oraram por sua saúde e os que estavam cientes das intercessões feitas por terceiros em intenção à sua saúde experimentaram melhorias na qualidade de vida relacionadas à saúde ao longo do tempo.^96^ No Multiethnic Study of Atherosclerosis (MESA), não foram observados padrões consistentes de associação entre as medidas de religiosidade e presença/extensão de DCV subclínica na avaliação basal ou incidentes dela ao longo de 4 anos.^97^ Com relação à associação entre espiritualidade/religiosidade e fatores de risco CV, os achados são discordantes. No estudo Women’s Health Initiative, envolvendo mais de 43.000 mulheres em menopausa, o risco CV mostrou-se maior em pacientes com atividade espiritual privada como orações, leitura da Bíblia e meditação.^98^ No Japão, em coorte com 36.965 indivíduos, os mais religiosos tiveram uma probabilidade significativamente maior de ter hábitos de saúde melhores e menos fatores de risco CV, exceto por uma maior prevalência de sobrepeso/obesidade na linha de base. Religiosidade também esteve associada a hábitos de saúde melhores ao longo do tempo e menor probabilidade de desenvolver diabetes, mas não de desenvolver HA ou dislipidemia.^99^

**Mensagens principais**

**Table:** 

**Risco de doença arterial coronariana e aspectos relacionados à espiritualidade/religiosidade**
No estudo InterHeart, os fatores de risco psicossociais (estresse e depressão) foram responsáveis por 33% do risco atribuível da população associado a infarto agudo do miocárdio.
**Risco de acidente vascular encefálico e aspectos relacionados à espiritualidade/religiosidade**
No estudo InterStroke, observou-se que os fatores psicossociais, (estresse e depressão) foram responsáveis por 17% do risco atribuível da população associado ao acidente vascular encefálico.
**Aspectos práticos**
Embora os resultados não sejam de consenso quanto aos benefícios da espiritualidade/religiosidade sobre a saúde cardiovascular, alguns estudos mostraram redução de eventos cardiovasculares e mortalidade naqueles indivíduos que tiveram práticas relacionadas à espiritualidade/religiosidade.

## Perspectivas, Lacunas do Conhecimento e Conclusão

Os conceitos envolvendo a medicina baseada em evidências (MBE) têm sido gradativamente aplicados também no domínio da pesquisa em espiritualidade e Doenças Cardiovasculares. As evidências científicas disponíveis podem ser avaliadas como promissoras, havendo ainda necessidade de pesquisas robustas com poder estatístico adequado, envolvendo estudos observacionais e aleatorizados, para que o promissor seja considerado comprovado, por meio de benefícios em desfechos clinicamente relevantes. Dessa maneira, evidências científicas em espiritualidade e medicina cardiovascular poderão influenciar e modificar a prática clínica e o prognóstico dos pacientes.

Nos mais diversos cenários discutidos nesse posicionamento sobre HA e Espiritualidade, foram buscados os dados da literatura disponíveis nessa área do conhecimento que ganham força e robustez. Espiritualidade é uma abordagem que deve ser feita atendendo a critérios bem-definidos de ação entre o profissional da saúde e os pacientes.

Do ponto de vista estrito das condutas definidas pela MBE, não encontraremos estudos com graus de recomendações I e nível de evidência A (condições para as quais há evidências conclusivas e o procedimento é seguro, e útil/eficaz a partir de dados obtidos de múltiplos estudos aleatorizados de bom porte, concordantes e/ou de metanálise robusta de estudos clínicos aleatorizados). Contudo, não trabalhamos com condições definidas por recomendações III e nível de evidência C (condições para as quais há evidências e/ou consenso de que o procedimento não é útil/eficaz e, em alguns casos, pode ser prejudicial e com dados obtidos de opiniões consensuais de especialistas).

O importante é a orientação do tema com respaldo científico, objetivando oferecer ao médico a possibilidade de ampliar a sua atuação e, ao paciente, a chance de que possa ter mais do que as indispensáveis e tradicionais abordagens da sua doença, enfermidade e vida.

Assim, é nosso dever oferecer o mais pleno sentido da saúde entendida como bem-estar físico, social, emocional, psíquico e espiritual, com base também nos conhecimentos e práticas desses conceitos sobre espiritualidade, aplicando-os com parcimônia e sensibilidade a quem nos é destinado cuidar.

## References

[1] 1. Lucchese FA, Koenig HG. Religion, spirituality and cardiovascular disease: research, clinical implications, and opportunities in Brazil. Rev Bras Cir Cardiovasc. 2013;28(1):103-28. doi: 10.5935/1678-9741.20130015. 10.5935/1678-9741.20130015 23739939

[2] 2. Lindeman M, Blomqvist S, Takada M. Distinguishing spirituality from other constructs: not a matter of well-being but of belief in supernatural spirits. J Nerv Ment Dis. 2012;200(2):167-73. doi: 10.1097/NMD.0b013e3182439719. 10.1097/NMD.0b013e3182439719 22297316

[3] 3. Précoma DB, Oliveira GMM, Simão AF, Dutra OP, Coelho OR, Izar MCO, et al. Updated Cardiovascular Prevention Guideline of the Brazilian Society of Cardiology – 2019. Arq Bras Cardiol. 2019;113(4):787-891. doi: 10.5935/abc.20190204. 10.5935/abc.20190204 PMC7020870 31691761

[4] 4. Koenig HG, Pargament KI, Nielsen J. Religious coping and health status in medically ill hospitalized older adults. J Nerv Ment Dis. 1998;186(9):513-21. doi: 10.1097/00005053-199809000-00001. 10.1097/00005053-199809000-00001 9741556

[5] 5. Steinhauser KE, Fitchett G, Handzo GF, Johnson KS, Koenig HG, Pargament KI, et al. State of the Science of Spirituality and Palliative Care Research Part I: definitions, measurement, and outcomes. J Pain Symptom Manage. 2017;54(3):428-40. doi: 10.1016/j.jpainsymman.2017.07.028. 10.1016/j.jpainsymman.2017.07.028 28733252

[6] 6. Borneman T, Ferrell B, Puchalski CM. Evaluation of the FICA tool for spiritual assessment. J Pain Symptom Manage. 2010;40(2):163-73. doi: 10.1016/j.jpainsymman.2009.12.019. 10.1016/j.jpainsymman.2009.12.019 20619602

[7] 7. Hummer RA, Ellison CG, Rogers RG, Moulton BE, Romero RR. Religious involvement and adult mortality in the United States: review and perspective. South Med J. 2004;97(12):1223-30. doi: 10.1097/01.SMJ.0000146547.03382.94. 10.1097/01.SMJ.0000146547.03382.94 15646761

[8] 8. Goldbourt U, Yaari S, Medalie JH. Factors predictive of long-term coronary heart disease mortality among 10,059 male Israeli civil servants and municipal employees. A 23-year mortality follow-up in the Israeli Ischemic Heart Disease Study. Cardiology. 1993;82(2-3):100-21. doi: 10.1159/000175862. 10.1159/000175862 8324774

[9] 9. Kristeller JL, Rhodes M, Cripe LD, Sheets V. Oncologist Assisted Spiritual Intervention Study (OASIS): patient acceptability and initial evidence of effects. Int J Psychiatry Med. 2005;35(4):329-47. doi: 10.2190/8AE4-F01C-60M0-85C8. 10.2190/8AE4-F01C-60M0-85C8 16673834

[10] 10. McCord G, Gilchrist VJ, Grossman SD, King BD, McCormick KE, Oprandi AM, et al. Discussing spirituality with patients: a rational and ethical approach. Ann Fam Med. 2004;2(4):356-61. doi: 10.1370/afm.71. 10.1370/afm.71 PMC1466687 15335136

[11] 11. Puchalski C, Ferrell B, Virani R, Otis-Green S, Baird P, Bull J, et al. Improving the quality of spiritual care as a dimension of palliative care: the report of the Consensus Conference. J Palliat Med. 2009;12(10):885-904. doi: 10.1089/jpm.2009.0142. 10.1089/jpm.2009.0142 19807235

[12] 12. Koenig HG. Taking a spiritual history. JAMA. 2004;291(23):2881-2. doi: 10.1001/jama.291.23.2881. 10.1001/jama.291.23.2881 15199042

[13] 13. Kliewer S. Allowing spirituality into the healing process. J Fam Pract. 2004;53(8):616-24. 15298831

[14] 14. Lucchetti G, Lucchetti AL, Vallada H. Measuring spirituality and religiosity in clinical research: a systematic review of instruments available in the portuguese language. Sao Paulo Med J. 2013;131(2):112-22. doi: 10.1590/s1516-31802013000100022. 10.1590/s1516-31802013000100022 PMC10871731 23657514

[15] 15. Silvestri GA, Knittig S, Zoller JS, Nietert PJ. Importance of faith on medical decisions regarding cancer care. J Clin Oncol. 2003;21(7):1379-82. doi: 10.1200/JCO.2003.08.036. 10.1200/JCO.2003.08.036 12663730

[16] 16. Balboni TA, Fitchett G, Handzo GF, Johnson KS, Koenig HG, Pargament KI, et al. State of the Science of Spirituality and Palliative Care Research Part II: screening, assessment, and interventions. J Pain Symptom Manage. 2017;54(3):441-53. doi: 10.1016/j.jpainsymman.2017.07.029. 10.1016/j.jpainsymman.2017.07.029 28734881

[17] 17. Fitchett G, Risk JL. Screening for spiritual struggle. J Pastoral Care Counsel. 2009;63(1-2):4-12. 20196352

[18] 18. Steinhauser KE, Voils CI, Clipp EC, Bosworth HB, Christakis NA, Tulsky JA. “Are you at peace?”: one item to probe spiritual concerns at the end of life. Arch Intern Med. 2006;166(1):101-5. doi: 10.1001/archinte.166.1.101. 10.1001/archinte.166.1.101 16401817

[19] 19. Mako C, Galek K, Poppito SR. Spiritual pain among patients with advanced cancer in palliative care. J Palliat Med. 2006;9(5):1106-13. doi: 10.1089/jpm.2006.9.1106. 10.1089/jpm.2006.9.1106 17040148

[20] 20. Berg G. The relationship between spiritual distress, PTSD and depression in Vietnam combat veterans. J Pastoral Care Counsel. 2011;65(1-2):6:1-11. doi: 10.1177/154230501106500106. 10.1177/154230501106500106 21919327

[21] 21. Koenig HG, Büssing A. The Duke University Religion Index (DUREL): a five-item measure for use in epidemiological studies. Religions. 2010;1(1):78-85. doi: 10.3390/rel1010078. 10.3390/rel1010078

[22] 22. Taunay TCE, Gondim FAA, Macêdo DS, Moreira-Almeida A; Gurgel LA, Andrade LMS, et al. Validação da versão brasileira da escala de religiosidade de Duke (DUREL). Rev Psiquiatr Clin 2012;39(4):130-5. doi: 10.1590/S0101-60832012000400003. 10.1590/S0101-60832012000400003

[23] 23. Puchalski C, Romer AL. Taking a spiritual history allows clinicians to understand patients more fully. J Palliat Med. 2000;3(1):129-37. doi: 10.1089/jpm.2000.3.129. 10.1089/jpm.2000.3.129 15859737

[24] 24. Neely D, Minford E. FAITH: spiritual history-taking made easy. Clin Teach. 2009;6(3):181-5.

[25] 25. Maugans TA. The spiritual history. Arch Fam Med. 1996;5:11-16 10.1001/archfami.5.1.11 8542049

[26] 26. Anandarajah G, Hight E. Spirituality and medical practice: using the HOPE questions as a practical tool for spiritual assessment. American family physician. 2001;63(1):81-9. doi: 10.1111/j.1743-498X.2009.00317.x 10.1111/j.1743-498X.2009.00317x 11195773

[27] 27. Lucchetti G, Granero AL, Bassi RM, Latorraca R, Nacif SAP. Espiritualidade na prática clínica: o que o clínico deve saber? Rev Bras Clin Med. 2010;8(2):154-8.

[28] 28. Lucchetti G, Bassi RM, Lucchetti ALG. Taking spiritual history in clinical practice: a systematic review of instruments. Explore. 2013;9(3):159-70. doi: 10.1016/j.explore.2013.02.004. 10.1016/j.explore.2013.02.004 23643371

[29] 29. Barroso WKS, Rodrigues CIS, Bortolotto LA, Mota-Gomes MA, Brandão AA, Feitosa ADM, et al. Brazilian Guidelines of Hypertension - 2020. Arq Bras Cardiol. 2021;116(3):516-658. doi: 10.36660/abc.2020123. 10.36660/abc.2020123 PMC9949730 33909761

[30] 30. Kaplan NM. Primary hypertension: pathogenesis. In: Kaplan NM, Victor RG, editors. Kaplan’s clinical hypertension. 11th. New York: Wolters Kluwer; 2015. p. 40-115.

[31] 31. Koenig HG. Religion, spirituality, and health: the research and clinical implications. ISRN Psychiatry. 2012;2012:278730. doi: 10.5402/2012/278730. 10.5402/2012/278730 PMC3671693 23762764

[32] 32. Mishra SK, Togneri E, Tripathi B, Trikamji B. Spirituality and Religiosity and Its Role in Health and Diseases. J Relig Health. 2017;56(4):1282-1301. doi: 10.1007/s10943-015-0100-z. 10.1007/s10943-015-0100-z 26345679

[33] 33. Cozier YC, Yu J, Wise LA, VanderWeele TJ, Balboni TA, Argentieri MA, et al. Religious and spiritual coping and risk of incident hypertension in the Black Women’s Health Study. Ann Behav Med. 2018;52(12):989-98. doi: 10.1093/abm/kay001. 10.1093/abm/kay001 PMC6230974 30418522

[34] 34. Ferraro KF, Kim S. Health benefits of religion among black and white older adults? Race, religiosity, and C-reactive protein. Soc Sci Med. 2014;120:92-9. doi: 10.1016/j.socscimed.2014.08.030. 10.1016/j.socscimed.2014.08.030 25226450

[35] 35. Shattuck EC, Muehlenbein MP. Religiosity/Spirituality and physiological markers of Health. J Relig Health. 2020;59(2):1035-54. doi: 10.1007/s10943-018-0663-6. 10.1007/s10943-018-0663-6 29978269

[36] 36. Lucchetti G, Granero AL, Nobre F, Avezum A Jr. Influência da religiosidade e espiritualidade na hipertensão arterial sistêmica. Rev Bras Hipertens. 2010;17(3):186-8.

[37] 37. Buck AC, Williams DR, Musick MA, Sternthal MJ. An examination of the relationship between multiple dimensions of religiosity, blood pressure, and hypertension. Soc Sci Med. 2009;68(2):314-22. doi: 10.1016/j.socscimed.2008.10.010. 10.1016/j.socscimed.2008.10.010 PMC2654362 19019516

[38] 38. Fitchett G, Powell LH. Daily spiritual experiences, systolic blood pressure, and hypertension among midlife women in SWAN. Ann Behav Med. 2009;37(3):257-67. doi: 10.1007/s12160-009-9110-y. 10.1007/s12160-009-9110-y PMC2867660 19662465

[39] 39. Koenig HG, George LK, Hays JC, Larson DB, Cohen HJ, Blazer DG. The relationship between religious activities and blood pressure in older adults. Int J Psychiatry Med. 1998;28(2):189-213. doi: 10.2190/75JM-J234-5JKN-4DQD. 10.2190/75JM-J234-5JKN-4DQD 9724889

[40] 40. Silva LB, Silva SS, Marcílio AG, Pierin AM. Prevalence of arterial hypertension among Seventh-Day Adventists of the São Paulo state capital and inner area. Arq Bras Cardiol. 2012;98(4):329-37. doi: 10.1590/s0066-782x2012005000026. 10.1590/s0066-782x2012005000026 22426989

[41] 41. Holt-Lunstad J, Steffen PR, Sandberg J, Jensen B. Understanding the connection between spiritual well-being and physical health: an examination of ambulatory blood pressure, inflammation, blood lipids and fasting glucose. J Behav Med. 2011;34(6):477-88. doi: 10.1007/s10865-011-9343-7. 10.1007/s10865-011-9343-7 21487720

[42] 42. Gillum RF, Ingram DD. Frequency of attendance at religious services, hypertension, and blood pressure: the Third National Health and Nutrition Examination Survey. Psychosom Med. 2006;68(3):382-5. doi: 10.1097/01.psy.0000221253.90559.dd. 10.1097/01.psy.0000221253.90559.dd 16738068

[43] 43. Bell CN, Bowie JV, Thorpe RJ Jr. The interrelationship between hypertension and blood pressure, attendance at religious services, and race/ethnicity. J Relig Health. 2012;51(2):310-22. doi: 10.1007/s10943-010-9346-7. 10.1007/s10943-010-9346-7 PMC3374060 20354789

[44] 44. Meng Q, Xu Y, Shi R, Zhang X, Wang S, Liu K, et al. Effect of religion on hypertension in adult Buddhists and residents in China: a cross-sectional study. Sci Rep. 2018;8(1):8203. doi: 10.1038/s41598-018-26638-4. 10.1038/s41598-018-26638-4 PMC5974409 29844414

[45] 45. Timio M, Lippi G, Venanzi S, Gentili S, Quintaliani G, Verdura C, et al. Blood pressure trend and cardiovascular events in nuns in a secluded order: a 30-year follow-up study. Blood Press. 1997;6(2):81-7. doi: 10.3109/08037059709061804. 10.3109/08037059709061804 9105646

[46] 46. Sethness R, Rauschhuber M, Etnyre A, Gilliland I, Lowry J, Jones ME. Cardiac health: relationships among hostility, spirituality, and health risk. J Nurs Care Qual. 2005;20(1):81-9. doi: 10.1097/00001786-200501000-00013. 10.1097/00001786-200501000-00013 15686080

[47] 47. Charlemagne-Badal SJ, Lee JW. Intrinsic religiosity and hypertension among older North American Seventh-Day Adventists. J Relig Health. 2016;55(2):695-708. doi: 10.1007/s10943-015-0102-x. 10.1007/s10943-015-0102-x PMC6091876 26330373

[48] 48. Spence ND, Farvid MS, Warner ET, VanderWeele TJ, Tworoger SS, Argentieri MA, et al. Religious service attendance, religious coping, and risk of hypertension in women participating in the Nurses’ Health Study II. Am J Epidemiol. 2020;189(3):193-203. doi: 10.1093/aje/kwz222. 10.1093/aje/kwz222 PMC7217278 31595952

[49] 49. Steptoe A. Psychosocial factors in the development of hypertension. Ann Med. 2000;32(5):371-5. doi: 10.3109/0785389000899594. 10.3109/0785389000899594 10949069

[50] 50. Cuffee Y, Ogedegbe C, Williams NJ, Ogedegbe G, Schoenthaler A. Psychosocial risk factors for hypertension: an update of the literature. Curr Hypertens Rep. 2014;16(10):483. doi: 10.1007/s11906-014-0483-3. 10.1007/s11906-014-0483-3 PMC4163921 25139781

[51] 51. Casagrande M, Favieri F, Guarino A, Di Pace E, Langher V, Germanò G, et al. The Night effect of anger: relationship with nocturnal blood pressure dipping. Int J Environ Res Public Health. 2020;17(8):2705. doi: 10.3390/ijerph17082705. 10.3390/ijerph17082705 PMC7216280 32326399

[52] 52. Vögele C, Steptoe A. Anger inhibition and family history as modulators of cardiovascular responses to mental stress in adolescent boys. J Psychosom Res. 1993;37(5):503-14. doi: 10.1016/0022-3999(93)90006-2. 10.1016/0022-3999(93)90006-2 8350292

[53] 53. Schnall PL, Pieper C, Schwartz JE, Karasek PA, Schlussel Y, Devereux RB, et al. The relationship between ‘job strain’ workplace diastolic blood pressure, and left ventricular mass index. Results of a case-control study. JAMA 1990; 263(14):1929-35. doi: 10.1001/jama.1990.03440140055031. 10.1001/jama.1990.03440140055031 2138234

[54] 54. Spruill TM. Chronic psychosocial stress and hypertension. Curr Hypertens Rep. 2010;12(1):10-6. doi: 10.1007/s11906-009-0084-8. 10.1007/s11906-009-0084-8 PMC3694268 20425153

[55] 55. Wiernik E, Nabi H, Pannier B, Czernichow S, Hanon O, Simon T, et al. Perceived stress, sex and occupational status interact to increase the risk of future high blood pressure: the IPC cohort study. J Hypertens. 2014;32(10):1979-86. doi: 10.1097/HJH.0000000000000288. 10.1097/HJH.0000000000000288 PMC5397536 24999800

[56] 56. Cozier Y, Palmer JR, Horton NJ, Fredman L, Wise LA, Rosenberg L. Racial discrimination and the incidence of hypertension in US black women. Ann Epidemiol. 2006;16(9):681-7. doi: 10.1016/j.annepidem.2005.11.008. 10.1016/j.annepidem.2005.11.008 16458539

[57] 57. Sparrenberger F, Cichelero FT, Ascoli AM, Fonseca FP, Weiss G, Berwanger O, et al. Does psychosocial stress cause hypertension? A systematic review of observational studies. J Hum Hypertens. 2009;23(1):12-9. doi: 10.1038/jhh.2008.74. 10.1038/jhh.2008.74 18615099

[58] 58. Gasperin D, Netuveli G, Dias-da-Costa JS, Pattussi MP. Effect of psychological stress on blood pressure increase: a meta-analysis of cohort studies. Cad Saude Publica. 2009;25(4):715-26. doi: 10.1590/s0102-311x2009000400002. 10.1590/s0102-311x2009000400002 19347197

[59] 59. Nabi H, Chastang JF, Lefèvre T, Dugravot A, Melchior M, Marmot MG, et al. Trajectories of depressive episodes and hypertension over 24 years: the Whitehall II prospective cohort study. Hypertension. 2011;57(4):710-6. doi: 10.1161/HYPERTENSIONAHA.110.164061. 10.1161/HYPERTENSIONAHA.110.164061 PMC3065997 21339474

[60] 60. Gangwisch JE, Malaspina D, Posner K, Babiss LA, Heymsfield SB, Turner JB, et al. Insomnia and sleep duration as mediators of the relationship between depression and hypertension incidence. Am J Hypertens. 2010;23(1):62-9. doi: 10.1038/ajh.2009.202. 10.1038/ajh.2009.202 PMC4415616 19893498

[61] 61. Fernandez-Mendoza J, Vgontzas AN, Liao D, Shaffer ML, Vela-Bueno A, Basta M, et al. Insomnia with objective short sleep duration and incident hypertension: the Penn State Cohort. Hypertension. 2012;60(4):929-35. doi: 10.1161/HYPERTENSIONAHA.112.193268. 10.1161/HYPERTENSIONAHA.112.193268 PMC3679545 22892811

[62] 62. Williams B, Mancia G, Spiering W, AgabitiRosei E, Azizi M, Burnier M, et al. 2018 Practice Guidelines for the management of arterial hypertension of the European Society of Hypertension and the European Society of Cardiology: ESH/ESC Task Force for the Management of Arterial Hypertension: Erratum. J Hypertens. 2019;37(2):456. doi: 10.1097/HJH.0000000000002026. 10.1097/HJH.0000000000002026 30640882

[63] 63. VanderWeele TJ, Balboni TA, Koh HK. Health and spirituality. JAMA. 2017;318(6):519-20. doi: 10.1001/jama.2017.8136. 10.1001/jama.2017.8136 28750127

[64] 64. Badanta-Romero B, Diego-Cordero R, Rivilla-García E. Influence of religious and spiritual elements on adherence to pharmacological treatment. J Relig Health. 2018;57(5):1905-17. doi: 10.1007/s10943-018-0606-2. 10.1007/s10943-018-0606-2 29582335

[65] 65. Kretchy IA, Owusu-Daaku FT, Danquah SA. Mental health in hypertension: assessing symptoms of anxiety, depression and stress on anti-hypertensive medication adherence. Int J Ment Health Syst. 2014;8:25. doi: 10.1186/1752-4458-8-25. 10.1186/1752-4458-8-25 PMC4077111 24987456

[66] 66. Mishra, SK, Togneri E, Tripathi B, Trikamji B. Spirituality and religiosity and Its role in health and diseases. J Relig Health. 2017; 56:1282–1301. 10.1007/s10943-015-0100-z 26345679

[67] 67. Abel WM, Greer DB. Spiritual/Religious beliefs & medication adherence in black women with hypertension. J Christ Nurs. 2017;34(3):164-9. doi: 10.1097/CNJ.0000000000000333. 10.1097/CNJ.0000000000000333 27662182

[68] 68. Naewbood S, Sorajjakool S, Triamchaisri SK. The role of religion in relation to blood pressure control among a Southern California Thai population with hypertension. J Relig Health. 2012;51(1):187-97. doi: 10.1007/s10943-010-9341-z. 10.1007/s10943-010-9341-z 20232251

[69] 69. Lewis LM, Ogedegbe G. Understanding the nature and role of spirituality in relation to medication adherence: a proposed conceptual model. Holist Nurs Pract. 2008;22(5):261-7. doi: 10.1097/01.HNP.0000334919.39057.14. 10.1097/01.HNP.0000334919.39057.14 PMC4755505 18758275

[70] 70. Silva CF, Borges RF, Avelino CCV, Miarelli AVTC, Vieira GIA, Goyatá SLT. Spirituality and religiosity in patients with systemic arterial hypertension. Rev Bioet. 2016;24(2):332-343. doi: 10.1590/1983-80422016242134 . 10.1590/1983-80422016242134

[71] 71. Marchiori MFR, Kozasa EH, Miranda RD, Andrade ALM, Perrotti TC, Leite JR. Decrease in blood pressure and improved psychological aspects through meditation training in hypertensive older adults: a randomized control study. Geriatr Gerontol Int. 2015;15(10):1158-64. doi: 10.1111/ggi.12414. 10.1111/ggi.12414 25407688

[72] 72. Levine GN, Lange RA, Bairey-Merz CN, Davidson RJ, Jamerson K, Mehta PK, et al. Meditation and cardiovascular risk reduction: a scientific statement from the American Heart Association. J Am Heart Assoc. 2017;6(10):e002218. doi: 10.1161/JAHA.117.002218. 10.1161/JAHA.117.002218 PMC5721815 28963100

[73] 73. Ooi SL, Giovino M, Pak SC. Transcendental meditation for lowering blood pressure: an overview of systematic reviews and meta-analyses. Complement Ther Med. 2017;34:26-34. doi: 10.1016/j.ctim.2017.07.008. 10.1016/j.ctim.2017.07.008 28917372

[74] 74. Anderson JW, Liu C, Kryscio RJ. Blood pressure response to transcendental meditation: a meta-analysis. Am J Hypertens. 2008;21(3):310-6. doi: 10.1038/ajh.2007.65. 10.1038/ajh.2007.65 18311126

[75] 75. Schneider RH, Grim CE, Rainforth MV, Kotchen T, Nidich SI, Gaylord-King C, et al. Stress reduction in the secondary prevention of cardiovascular disease: randomized, controlled trial of transcendental meditation and health education in blacks. Circ Cardiovasc Qual Outcomes. 2012;5(6):750-8. doi: 10.1161/CIRCOUTCOMES.112.967406. 10.1161/CIRCOUTCOMES.112.967406 PMC7269100 23149426

[76] 76. Hagins M, States R, Selfe T, Innes K. Effectiveness of yoga for hypertension: systematic review and meta-analysis. Evid Based Complement Alternat Med. 2013;2013:649836. doi: 10.1155/2013/649836. 10.1155/2013/649836 PMC3679769 23781266

[77] 77. Koenig HG, King DE, Carson VB. Handbook of religion and health. 2nd ed. London: Oxford University Press; 2012.

[78] 78. Sanchez SA, Chung C, Mejia A, Ramirez FE, Shavlik GW, Bivens RL, et al. Multiple lifestyle interventions reverses hypertension. Cogent Med. 2019; 6(1):1-13. doi: 10.1080/2331205X.2019.1636534. 10.1080/2331205X.2019.1636534

[79] 79. Albargawi M, Snethen J, Al Gannass A, Kelber S. Relationship between person’s health beliefs and diabetes self-care management regimen. J Vasc Nurs. 2017;35(4):187-92. doi: 10.1016/j.jvn.2017.07.002. 10.1016/j.jvn.2017.07.002 29153226

[80] 80. Black G, Davis BA, Heathcotte K, Mitchell N, Sanderson C. The relationship between spirituality and compliance in patients with heart failure. Prog Cardiovasc Nurs. 2006;21(3):128-33. doi: 10.1111/j.0889-7204.2006.04804.x. 10.1111/j.0889-7204.2006.04804.x 16957458

[81] 81. Alvarez JS, Goldraich LA, Nunes AH, Zandavalli MC, Zandavalli RB, Belli KC, et al. Association between spirituality and adherence to management in outpatients with heart failure. Arq Bras Cardiol. 2016;106(6):491-501. doi: 10.5935/abc.20160076. 10.5935/abc.20160076 PMC4940148 27192385

[82] 82. Saffari M, Lin CY, Chen H, Pakpour AH. The role of religious coping and social support on medication adherence and quality of life among the elderly with type 2 diabetes. Qual Life Res. 2019;28(8):2183-93. doi: 10.1007/s11136-019-02183-z. 10.1007/s11136-019-02183-z 31037591

[83] 83. Lewis LM. Medication adherence and spiritual perspectives among african american older women with hypertension. A qualitative study. J Gerontol Nurs. 2011;37(6):34-41. doi: 10.3928/00989134-20100201-02. 10.3928/00989134-20100201-02 21323238

[84] 84. Abel WM, Joyner JS, Cornelius JB, Greer DB. Self-care management strategies used by black women who self-report consistent adherence to antihypertensive medication. Patient Prefer Adherence. 2017;11:1401-12. doi: 10.2147/PPA.S138162. 10.2147/PPA.S138162 PMC5565386 28860723

[85] 85. Kretchy I, Owusu-Daaku F, Danquah S. Spiritual and religious beliefs: do they matter in the medication adherence behaviour of hypertensive patients? Biopsychosoc Med. 2013;7(1):15. doi: 10.1186/1751-0759-7-15. 10.1186/1751-0759-7-15 PMC3854617 24138844

[86] 86. Abel WM, Efird JT. The association between trust in health care providers and medication adherence among black women with hypertension. Front Public Health. 2013;1:66. doi: 10.3389/fpubh.2013.00066. 10.3389/fpubh.2013.00066 PMC3860006 24350234

[87] 87. Shahin W, Kennedy GA, Stupans I. The impact of personal and cultural beliefs on medication adherence of patients with chronic illnesses: a systematic review. Patient Prefer Adherence. 2019;13:1019-35. doi: 10.2147/PPA.S212046. 10.2147/PPA.S212046 PMC6611718 31303749

[88] 88. Lapane KL, Lasater TM, Allan C, Carleton RA. Religion and cardiovascular disease risk. J Rel Health. 1997;36:155-64. doi: 10.1023/A:1027444621177. 10.1023/A:1027444621177

[89] 89. Yusuf S, Hawken S, Ounpuu S, Dans T, Avezum A, Lanas F, et al. Effect of potentially modifiable risk factors associated with myocardial infarction in 52 countries (the INTERHEART study): case-control study. Lancet. 2004;364(9438):937-52. doi: 10.1016/S0140-6736(04)17018-9. 10.1016/S0140-6736(04)17018-9 15364185

[90] 90. O’Donnell MJ, Chin SL, Rangarajan S, Xavier D, Liu L, Zhang H, et al. Global and regional effects of potentially modifiable risk factors associated with acute stroke in 32 countries (INTERSTROKE): a case-control study. Lancet. 2016;388(10046):761-75. doi: 10.1016/S0140-6736(16)30506-2. 10.1016/S0140-6736(16)30506-2 27431356

[91] 91. McCullough ME, Hoyt WT, Larson DB, Koenig HG, Thoresen C. Religious involvement and mortality: a meta-analytic review. Health Psychol. 2000;19(3):211-22. doi: 10.1037//0278-6133.19.3.211. 10.1037//0278-6133.19.3.211 10868765

[92] 92. Chida Y, Steptoe A, Powell LH. Religiosity/spirituality and mortality. A systematic quantitative review. Psychother Psychosom. 2009;78(2):81-90. doi: 10.1159/000190791. 10.1159/000190791 19142047

[93] 93. VanderWeele TJ, Yu J, Cozier YC, Wise L, Argentieri MA, Rosenberg L, et al. Attendance at religious services, prayer, religious coping, and religious/spiritual identity as predictors of all-cause mortality in the black women’s health study. Am J Epidemiol. 2017;185(7):515-22. doi: 10.1093/aje/kww179. 10.1093/aje/kww179 PMC6354668 28338863

[94] 94. Li S, Stampfer MJ, Williams DR, VanderWeele TJ. Association of religious service attendance with mortality among women. JAMA Intern Med. 2016;176(6):777-85. doi: 10.1001/jamainternmed.2016.1615. 10.1001/jamainternmed.2016.1615 PMC5503841 27183175

[95] 95. Abu HO, Lapane KL, Waring ME, Ulbricht CM, Devereaux RS, McManus DD, et al. Religious practices and long-term survival after hospital discharge for an acute coronary syndrome. PLoS One. 2019;14(10):e0223442. doi: 10.1371/journal.pone.0223442. 10.1371/journal.pone.0223442 PMC6777785 31584980

[96] 96. Abu HO, McManus DD, Lessard DM, Kiefe CI, Goldberg RJ. Religious practices and changes in health-related quality of life after hospital discharge for an acute coronary syndrome. Health Qual Life Outcomes. 2019;17(1):149. doi: 10.1186/s12955-019-1218-6. 10.1186/s12955-019-1218-6 PMC6724337 31481073

[97] 97. Feinstein M, Liu K, Ning H, Fitchett G, Lloyd-Jones DM. Burden of cardiovascular risk factors, subclinical atherosclerosis, and incident cardiovascular events across dimensions of religiosity: the multi-ethnic study of atherosclerosis. Circulation. 2010;121(5):659-66. doi: 10.1161/CIRCULATIONAHA.109.879973. 10.1161/CIRCULATIONAHA.109.879973 PMC2871276 20100975

[98] 98. Salmoirago-Blotcher E, Fitchett G, Hovey KM, Schnall E, Thomson C, Andrews CA, et al. Frequency of private spiritual activity and cardiovascular risk in postmenopausal women: the women’s health initiative. Ann Epidemiol. 2013;23(5):239-45. doi: 10.1016/j.annepidem.2013.03.002. 10.1016/j.annepidem.2013.03.002 PMC3741666 23621989

[99] 99. Kobayashi D, Shimbo T, Takahashi O, Davis RB, Wee CC. The relationship between religiosity and cardiovascular risk factors in Japan: a large-scale cohort study. J Am Soc Hypertens. 2015;9(7):553-62. doi: 10.1016/j.jash.2015.04.003. 10.1016/j.jash.2015.04.003 PMC4597307 26188400

