# Supplementary material for: Posicionamento sobre Hipertensão Arterial e Espiritualidade – 2021
Source: Arq Bras Cardiol. 2021 Sep 1;117(3):599–613. [Article in Portuguese] doi: 10.36660/abc.20210723 (PMC8462965; doi:10.36660/abc.20210723)
Supplement: Translation

# Translation: Position Statement on Hypertension and Spirituality – 2021

**Authors:** Fernando Nobre, Roberto Esporcatte, Andréa Araujo Brandão, Álvaro Avezum, Audes Diógenes Magalhães Feitosa, Celso Amodeo, Eduardo Costa Duarte Barbosa, Emilio Hideyuki Moriguchi, Fernando Antônio Lucchese, Hermilo Borba Griz, José Carlos Nicolau, Lucélia Batista Neves Cunha Magalhães, Marco Antônio Mota-Gomes, Mario Henrique Elesbão de Borba, Mauro Ricardo Nunes Pontes, Paulo César Brandão Veiga Jardim, Pedro Pimenta de Mello Spineti, Ricardo Mourilhe-Rocha, Roberto Dischinger Miranda, Sérgio Lívio Menezes Couceiro, Weimar Kunz Sebba Barroso

**Affiliations:** 1 Ribeirão Preto SP Brazil Hospital das Clínicas da Faculdade de Medicina de Ribeirão Preto da Universidade de São Paulo, Ribeirão Preto, SP – Brazil; 2 Ribeirão Preto SP Brazil Hospital São Francisco, Ribeirão Preto, SP – Brazil; 3 Rio de Janeiro RJ Brazil Universidade do Estado do Rio de Janeiro (UERJ), Rio de Janeiro, RJ – Brazil; 4 Rio de Janeiro RJ Brazil Hospital Pró-Cradíaco, Rio de Janeiro, RJ – Brazil; 5 São Paulo SP Brazil Hospital Alemão Oswaldo Cruz, São Paulo, SP – Brazil; 6 Recife PE Brazil Universidade Federal de Pernambuco, Recife, PE – Brazil; 7 Recife PE Brazil Pronto Socorro Cardiológico de Pernambuco (PROCAPE), Recife, PE – Brazil; 8 Recife SP Brazil Universidade Federal de São Paulo (UNIFESP), São Paulo, SP – Brazil; 9 Porto Alegre RS Brazil Santa Casa de Misericórdia de Porto Alegre, Porto Alegre, RS – Brazil; 10 Porto Alegre RS Brazil Universidade Federal do Rio Grande do Sul, Porto Alegre, RS – Brazil; 11 Recife PE Brazil Hospital Agamenon Magalhães, Recife, PE – Brazil; 12 São Paulo SP Brazil Instituto do Coração (InCor), Hospital das Clínicas da Faculdade de Medicina da Universidade de São Paulo (HCFMUSP), São Paulo, SP – Brazil; 13 Salvador BA Brazil Centro Universitário de Tecnologia e Ciência (UniFTC), Salvador, BA – Brazil; 14 Maceió AL Brazil Centro Universitário CESMAC, Maceió, AL – Brazil; 15 Lajeado RS Brazil Cardio Clínica do Vale, Lajeado, RS – Brazil; 16 Goiânia GO Brazil Liga de Hipertensão Arterial, Goiânia, GO – Brazil; 17 Goiânia GO Brazil Hospital do Coração de Goiás, Goiânia, GO – Brazil; 18 Cabo Frio RJ Brazil Hospital Santa Izabel, Cabo Frio, RJ – Brazil; 19 Goiânia GO Brazil Universidade Federal de Goiás, Goiânia, GO – Brazil

**Table:** 

Position Statement on Hypertension and Spirituality – 2021	
The report below lists declarations of interest as reported to the SBC by the experts during the period of the development of these statement, 2020/2021.	
Expert	Type of relationship with industry
Álvaro Avezum Jr.	Funding of continuing medical education activities, including travel, accommodation and registration in conferences and courses, from the brazilian or international pharmaceutical, orthosis, prosthesis, equipment and implants industry:
	- EMS: SBC Spirituality Symposia - DEMCA.
Andréa Araujo Brandão	Financial declaration
	A - Economically relevant payments of any kind made to (i) you, (ii) your spouse/partner or any other person living with you, (iii) any legal person in which any of these is either a direct or indirect controlling owner, business partner, shareholder or participant; any payments received for lectures, lessons, training instruction, compensation, fees paid for participation in advisory boards, investigative boards or other committees, etc. From the brazilian or international pharmaceutical, orthosis, prosthesis, equipment and implants industry:
	- Servier, Libbs e Merck: Hypertension.
Audes Diógenes Magalhães Feitosa	Funding of continuing medical education activities, including travel, accommodation and registration in conferences and courses, from the brazilian or international pharmaceutical, orthosis, prosthesis, equipment and implants industry:
	- EMS, Servier, Sandoz, Merck, Medtronic, Omron.
Celso Amodeo	Nothing to be declared
Eduardo Costa Duarte Barbosa	Financial declaration
	A - Economically relevant payments of any kind made to (i) you, (ii) your spouse/partner or any other person living with you, (iii) any legal person in which any of these is either a direct or indirect controlling owner, business partner, shareholder or participant; any payments received for lectures, lessons, training instruction, compensation, fees paid for participation in advisory boards, investigative boards or other committees, etc. From the brazilian or international pharmaceutical, orthosis, prosthesis, equipment and implants industry:
	- EMS, Servier: Antihypertensives; Cardios: Equipment.
	Other relationships
	Funding of continuing medical education activities, including travel, accommodation and registration in conferences and courses, from the brazilian or international pharmaceutical, orthosis, prosthesis, equipment and implants industry:
	- Servier: Antihypertensives.
Emilio Hideyuki Moriguchi	Financial declaration
	A - Economically relevant payments of any kind made to (i) you, (ii) your spouse/partner or any other person living with you, (iii) any legal person in which any of these is either a direct or indirect controlling owner, business partner, shareholder or participant; any payments received for lectures, lessons, training instruction, compensation, fees paid for participation in advisory boards, investigative boards or other committees, etc. From the brazilian or international pharmaceutical, orthosis, prosthesis, equipment and implants industry:
	- Amgen, AstraZeneca, Biolab, Kowa, MSD, Daiichi-Sankyo, Sanofi: Cardiology.
	B - Research funding under your direct/personal responsibility (directed to the department or institution) from the brazilian or international pharmaceutical, orthosis, prosthesis, equipment and implants industry:
	- Amgen, MSD, Daiichi-Sankyo, Kowa: Cardiology.
	Other relationships
	Funding of continuing medical education activities, including travel, accommodation and registration in conferences and courses, from the brazilian or international pharmaceutical, orthosis, prosthesis, equipment and implants industry:
	- Amgen, Biolab, Kowa, MSD, Daiichi-Sankyo, Sanofi: Cardiology.
Fernando Antônio Lucchese	Nothing to be declared
Fernando Nobre	Financial declaration
	A - Economically relevant payments of any kind made to (i) you, (ii) your spouse/partner or any other person living with you, (iii) any legal person in which any of these is either a direct or indirect controlling owner, business partner, shareholder or participant; any payments received for lectures, lessons, training instruction, compensation, fees paid for participation in advisory boards, investigative boards or other committees, etc. From the brazilian or international pharmaceutical, orthosis, prosthesis, equipment and implants industry:
	- Libbs, Novartis, Servier, Baldacci.
	Other relationships
	Funding of continuing medical education activities, including travel, accommodation and registration in conferences and courses, from the brazilian or international pharmaceutical, orthosis, prosthesis, equipment and implants industry:
	- Libbs, Cristália, Daichi Sankio, Novartis, Biolab, Servier, Baldacci.
Hermilo Borba Griz	Financial declaration
	A - Economically relevant payments of any kind made to (i) you, (ii) your spouse/partner or any other person living with you, (iii) any legal person in which any of these is either a direct or indirect controlling owner, business partner, shareholder or participant; any payments received for lectures, lessons, training instruction, compensation, fees paid for participation in advisory boards, investigative boards or other committees, etc. From the brazilian or international pharmaceutical, orthosis, prosthesis, equipment and implants industry:
	- Pfizer, Bayer: Anticoagulation.
José Carlos Nicolau	Financial declaration
	A - Economically relevant payments of any kind made to (i) you, (ii) your spouse/partner or any other person living with you, (iii) any legal person in which any of these is either a direct or indirect controlling owner, business partner, shareholder or participant; any payments received for lectures, lessons, training instruction, compensation, fees paid for participation in advisory boards, investigative boards or other committees, etc. From the brazilian or international pharmaceutical, orthosis, prosthesis, equipment and implants industry:
	- Amgen: Lipid-lowering; Bayer: Anticoagulant; Daiichi Sankyo: Antiplatelet, anticoagulant; Novartis: SRA inhibitor, lipid-lowering; Sanofi: Anticoagulant, lipid-lowering, antiplatelet; Servier: antiplatelet. B - Research funding under your direct/personal responsibility (directed to the department or institution) from the brazilian or international pharmaceutical, orthosis, prosthesis, equipment and implants industry:
	- Astrazeneca: Antiplatelet, hypoglycemic; Bayer: Anticoagulant; Esperion: Lipid-lowering; CSL Behring: lipid-lowering; Dalcor: HDL increase; Jansen: Anticoagulant; Novartis: SARS inhibitor; Novo Nordisk: Hypoglycemic; Sanofi: Anticoagulant, lipid-lowering; Vifor: Iron Deficiency.
	C - Personal research funding paid by the brazilian or international pharmaceutical, orthosis, prosthesis, equipment and implants industry:
	- Roche: Laboratory kits for platelet aggregability assessment.
	Other relationships
	Funding of continuing medical education activities, including travel, accommodation and registration in conferences and courses, from the brazilian or international pharmaceutical, orthosis, prosthesis, equipment and implants industry:
	- Sanofi: Anticoagulant, antiplatelet, lipid-lowering; Bayer: Anticoagulant; Servier: Antiplatelet.
Lucélia Batista Neves Cunha Magalhães	Nothing to be declared
Marco Antônio Mota-Gomes	Financial declaration
	A - Economically relevant payments of any kind made to (i) you, (ii) your spouse/partner or any other person living with you, (iii) any legal person in which any of these is either a direct or indirect controlling owner, business partner, shareholder or participant; any payments received for lectures, lessons, training instruction, compensation, fees paid for participation in advisory boards, investigative boards or other committees, etc. From the brazilian or international pharmaceutical, orthosis, prosthesis, equipment and implants industry:
	- Omron, Beliva, Libbs.
	Other relationships
	Funding of continuing medical education activities, including travel, accommodation and registration in conferences and courses, from the brazilian or international pharmaceutical, orthosis, prosthesis, equipment and implants industry:
	- Omron, Libbs.
Mario Henrique Elesbão de Borba	Nothing to be declared
Mauro Ricardo Nunes Pontes	Financial declaration
	A - Economically relevant payments of any kind made to (i) you, (ii) your spouse/partner or any other person living with you, (iii) any legal person in which any of these is either a direct or indirect controlling owner, business partner, shareholder or participant; any payments received for lectures, lessons, training instruction, compensation, fees paid for participation in advisory boards, investigative boards or other committees, etc. From the brazilian or international pharmaceutical, orthosis, prosthesis, equipment and implants industry:
	- Novo Nordisk: Ozempic; Mantecorp: NesinaPio.
	Other relationships
	Funding of continuing medical education activities, including travel, accommodation and registration in conferences and courses, from the brazilian or international pharmaceutical, orthosis, prosthesis, equipment and implants industry:
	- Servier: Acertanlo.
Paulo César Brandão Veiga Jardim	Nothing to be declared
Pedro Pimenta de Mello Spineti	Financial declaration
	B - Research funding under your direct/personal responsibility (directed to the department or institution) from the brazilian or international pharmaceutical, orthosis, prosthesis, equipment and implants industry:
	- Boehringer Ingelheim: Empaglifozina.
	Other relationships
	Funding of continuing medical education activities, including travel, accommodation and registration in conferences and courses, from the brazilian or international pharmaceutical, orthosis, prosthesis, equipment and implants industry:
	- Servier: Heart failure; Novartis: Entresto.
Ricardo Mourilhe-Rocha	Nothing to be declared
Roberto Dischinger Miranda	Funding of continuing medical education activities, including travel, accommodation and registration in conferences and courses, from the brazilian or international pharmaceutical, orthosis, prosthesis, equipment and implants industry:
	- EMS, Boehringher, Sanofi, Servier.
Roberto Esporcatte	Nothing to be declared
Sérgio Lívio Menezes Couceiro	Financial declaration
	A - Economically relevant payments of any kind made to (i) you, (ii) your spouse/partner or any other person living with you, (iii) any legal person in which any of these is either a direct or indirect controlling owner, business partner, shareholder or participant; any payments received for lectures, lessons, training instruction, compensation, fees paid for participation in advisory boards, investigative boards or other committees, etc. From the brazilian or international pharmaceutical, orthosis, prosthesis, equipment and implants industry:
	- Pfizer, AstraZeneca, Servier, Medley: Speaker and lectures.
	Other relationships
	Funding of continuing medical education activities, including travel, accommodation and registration in conferences and courses, from the brazilian or international pharmaceutical, orthosis, prosthesis, equipment and implants industry:
	- Aché, Pfizer, Servier, AstraZeneca: Lectures.
Weimar Kunz Sebba Barroso	Financial declaration
	A - Economically relevant payments of any kind made to (i) you, (ii) your spouse/partner or any other person living with you, (iii) any legal person in which any of these is either a direct or indirect controlling owner, business partner, shareholder or participant; any payments received for lectures, lessons, training instruction, compensation, fees paid for participation in advisory boards, investigative boards or other committees, etc. From the brazilian or international pharmaceutical, orthosis, prosthesis, equipment and implants industry:
	- EMS, Servier, Brace Pharma, Omron, Cardios: Hypertension.
	B - Research funding under your direct/personal responsibility (directed to the department or institution) from the brazilian or international pharmaceutical, orthosis, prosthesis, equipment and implants industry:
	- Ministério da Saúde, PROADI SUS: Hypertension; Einstein: Research projects.
	Other relationships
	Any economically relevant equity interest in companies in the healthcare or education industry or in any companies competing with or supplying to SBC:
	- Beliva Health.

## Basic Concepts about Spirituality and Forms of Evaluation

The definitions of spirituality and religiosity vary in different cultural and religious environments.^1^ There is a diversity of concepts without a clear definition, and the term is used inaccurately and inconsistently, which impedes measurement.^2^

The Brazilian Society of Cardiology’s Department of Spirituality Studies and Cardiovascular Medicine considers spirituality “a set of observable and measurable moral, mental. and emotional values that are motivated by the will and guide thoughts, behaviors, and attitudes in intra- and interpersonal relationships”.^3^ Spirituality should be valued and is applicable to all individuals, regardless of religious affiliation, including atheists, agnostics and those with no religious affiliation or who do not observe its precepts.^4^

Religiosity is the degree to which an individual believes in, follows and practices a religion. It can be organized (participation in church, temple, or religious services) or non-organized (praying, reading books or watching religious programs on one’s own initiative).^3^ Koenig describes religion as an “organized system of beliefs, practices, and symbols designed to facilitate closeness to the transcendent or the Divine and foster an understanding of one’s relationship and responsibilities with others living in community”.^1^^,^^5^ Religion is a multidimensional construct that includes beliefs, behaviors, dogmas, rituals, and ceremonies that can be performed or practiced privately or publicly, that is in some way derived from established traditions developed within a community.

Coping, or religious coping, is an important mechanism for understanding how religiosity/spirituality can affect health: it is the use of religiosity as a means of adapting to physical, psychological, and social challenges in diseases and health changes.

## Scales for Assessing Religiosity and Spirituality

Spirituality/religiosity can be assessed in the “spiritual history”, which is a set of questions that allows patients to share their spiritual and religious values, identifying possible spiritual issues that may interfere with the therapy. It is always necessary to focus on patients and be guided by what they reveal.^6^ This component of the medical history should be evaluated in all patients who seek medical care; spiritual anamnesis should also be performed with all inpatients and outpatients, especially in those with chronic diseases.

Many patients are religious/spiritual, and their beliefs help them cope with disease and face adverse life situations, although certain situations can involve points of conflict with medical practice. During periods of hospitalization or chronic illness, patients are removed from communities and are unable to practice their religion. In addition, their personal beliefs can affect health-related decisions, sometimes interfering with fundamental aspects of treatment.^7^^,^^8^

Studies show that most patients would like their doctors to ask about religiosity/spirituality and that doing so would lead to greater empathy and trust,^9^ which would result in more humanized care^10^ and greater participation by both parties.^7^^,^^8^

The objectives of evaluating religiosity/spirituality as a social and demographic factor include: understanding the patient’s beliefs, identifying aspects that might interfere with health care, assessing individual/social/family spiritual strength to cope with the disease, offer empathy and support, and help the patient accept the disease, as well as identifying conflict or suffering that requires evaluation by a trained professional.^11^^,^^12^ Although the topic can be approached in a number of ways, the most important point is to do so with sensitivity, without imposing or promoting specific beliefs or practices. Religion should not be prescribed, forced, or encouraged, so as not to add guilt to the disease burden. Common sense should be the rule. In extreme situations, such as serious accidents or acute myocardial infarction, discussing religion can lead to stress and even worsen the patient’s condition.^13^^,^^14^

Naturally, when the physician has not adequately prepared for this type of approach or when the patient refuses it, it should not be performed. For non-religious or adverse patients, it is necessary to understand how they face and live with the disease, what fosters purpose and meaning in their lives (family, friends, hobbies, etc.), and what cultural beliefs may impact their treatment.^15^

### Scales and Instruments

The various psychometric instruments can be divided into different categories:^16^^,^^17^

#### Spiritual Inventory

These brief and easy to apply measures are used to determine the presence of spiritual needs that require deeper evaluation (Chart 1).

**Chart 1:** Types of spiritual inventory

Tracking tools	Spiritual Domains Evaluated
"Rush" protocol for tracking religiosity/spirituality^17^	The importance of religiosity/spirituality in the disease; strength or spiritual comfort
“Are you at peace?”^18^	Inner peace
“Do you feel spiritual pain or suffering?”^19^	Spiritual pain/suffering
Spiritual Injury Scale^20^	Guilt, anger, sadness, feeling of injustice, fear of death

#### Instruments for Taking a Spiritual History

These instruments allow a broader assessment of the different domains of religiosity/spirituality that can affect clinical evolution, posture, self-care, and physical, mental, and spiritual well-being in the face of disease. They are well-structured and cover different domains, but they should be applied from memory, informally, throughout the conversation. They should serve as a guide, not being viewed rigidly but as a continuous learning process that is part of anamnesis. Several instruments have been validated for data collection, whether to assess religiosity/spirituality or for research purposes.

#### Religiosity Scales

The Duke University Religion Index (DUREL)^21^ is a five-item scale that measures three dimensions of religious involvement: The first item assesses organizational religiosity, the second assesses non-organizational religiosity, and items 3, 4 and 5 assess intrinsic religiosity. The DUREL is succinct and easy to apply, has been validated for use in Brazil^22^ and addresses the main areas of religiosity. The DUREL’s dimensions have been shown to be related to several indicators of social support and health.^22^

#### Instruments for Assessing Spiritual History

Based on previously validated scales, these instruments involve a set of questions in different domains that are associated with health outcomes. The main scales include: FICA,^23^ FAITH,^24^ SPIRIT,^25^ and HOPE.^26^ FICA, which was created by physicians and has shown the best psychometric properties, can be used in different situations. It assesses four dimensions (faith/beliefs, importance/influence, community, and address) and is easily applied and memorized.^27^

Even with complete respect for the patient’s beliefs and without proselytizing, religious issues can prove complex and involve conflict. They must be handled with empathy and understanding. When conflict is evident, a visit by the religious leader should be considered.

Once spiritual tools are identified, patients should be encouraged to use them as a way of preventing disease. Such practices, beyond prayer and meditation, can include reading, music, etc. Patients must be encouraged to find spiritual aspects that, together with standard treatment, can improve disease outcomes. In serious illnesses, physicians can help patients find meaning and acceptance by coping with the situation using their spiritual resources in the best possible way. Often, through such interactions, alternatives and adjustments to the therapeutic plan emerge in which patient autonomy and patient-centered care are strengthened.^27^^,^^28^

**Main messages**

**Table:** 

**Concepts**
**Spirituality** is a set of observable and measurable moral, mental, and emotional values that are motivated by the will and guide thoughts, behaviors and attitudes in intra- and interpersonal relationships.
**Religiosity** is how much an individual believes in, follows and practices a religion.
**Practical application**
If the professional and patient are willing and able, religiosity/spirituality should always be assessed.
The assessment must be performed through questionnaires developed for use by health professionals.
**How to proceed after obtaining the spiritual history**
Information about the spiritual dimension can broaden our understanding of the impact of religiosity/spirituality on the course of the disease and identify patient demands in this area.

## Mechanisms Involving Spirituality and the Pathophysiology of Arterial Hypertension

Arterial hypertension (AH) is characterized as a multifactorial and multigenic disease that mainly affects the vascular system (small and large arteries), leading to progressive and, after a certain point, irreversible damage to the so-called target organs (heart, kidneys and brain).^29^ Neural, hormonal, renal and vascular biological mechanisms participate and interact in different ways to result in sustained elevation of blood pressure (BP). Multiple factors are involved in and influence biological systems, such as: genetic, environmental, humoral, hemodynamic, neural, endocrinal, anatomical, and adaptive factors. More recently, inflammation and the production of reactive oxygen species have been implicated in several of these factors on cellular and molecular levels.^30^

The mechanisms involved in BP control depend on intrinsic and extrinsic stimuli. In situations of stress, the sympathetic nervous system is activated, which discharges vasoactive substances that facilitate the onset of AH.^30^ In situations of stress and anxiety, dietary errors, such as excessive intake of carbohydrates, fat and salt, also contribute to increased BP. Thus, human beings must be understood as biopsychosocial beings, and these dimensions are strongly interrelated. It is recognized that spirituality has an influence on this triad, participating in its balance.^3^

Long-term activation of the sympathetic nervous system, the renin-angiotensin-aldosterone system, altered renal mechanisms, sodium retention, and vascular alterations (represented by endothelial dysfunction and alterations of the vascular smooth muscle layer) are interrelated and are strongly implicated in the development and in the maintenance of AH through a sustained increase in peripheral vascular resistance, increased levels of inflammatory markers, and target organ damage.^30^

Studies have investigated the relationship between various dimensions of religiosity/spirituality and BP, and most show an association with lower BP values and lower rates of AH,^1^^,^^31^ especially reduced diastolic BP.^4^ It is possible that individuals who are more connected with religiosity/spirituality have more positive feelings (love, peace, forgiveness) than negative feelings (fear, anxiety, depression) and this aspect promotes reduced sympathetic nervous system activity and cortisol levels. However, the studies are not completely conclusive.^1^^,^^31^^,^^32^

For coping with stress, religiosity/spirituality has been associated with a lower incidence of AH, especially in the presence of higher stress levels^33^ and reduced C-reactive protein levels in African-American populations.^34^

A meta-analysis of 87 studies investigated the impact of religiosity/spirituality practices on physiological health markers, finding an inverse association between religiosity/spirituality measures, particularly participation in religious services, intrinsic religiosity, prayer, and meditation and lower stress, BP, and inflammatory markers (C-reactive protein), although the effect size was small and there was substantial heterogeneity among the studies, which suggests the presence of confounding factors.^35^

**Main messages**

**Table:** 

**Concepts**
Clinical studies investigating the relationship of religiosity/spirituality with blood pressure and biological markers are observational, with most suggesting an association with lower blood pressure values, lower levels of inflammatory markers, reduced sympathetic activity, and reduced cortisol levels.
**Practical aspects**
Spirituality/religiosity practices, especially participation in worship services, intrinsic religiosity, prayers, and meditation, are inversely associated with physiological markers, such as stress level, blood pressure, and inflammatory markers (C-reactive protein).

## The Association between Spirituality, High Blood Pressure, and Evidence in Primary Prevention

Although studies about the influence of psychological and religious/spiritual factors on high BP have provided further insight into the incidence and prevalence of AH, their results are not in agreement.^36^

Objectively measuring feelings such as forgiveness, optimism, pessimism, hostility, empathy, peaceful states, and the emotional stress involved in spirituality is difficult. An individual’s degree of spirituality can often be quantified by the frequency of activity in a religious community or time spent reading about themes in the chosen religion. Thus, following other studies, we will present evidence of religiosity/spirituality indistinctly at certain points.

The Chicago Community Adult Health Study found that a greater religiosity was not a protective factor against AH. However, lower diastolic BP values were found in individuals with a propensity for forgiveness, compared to those without it.^37^

Similarly, the Study of Women’s Health Across the Nation, which involved 1,658 participants and used the Daily Spiritual Experiences Scale, found no significant difference between daily experiences of spirituality and the prevalence/incidence of AH in 3 years of follow-up.^38^

In another population-based longitudinal cohort study of older adults, Koenig et al. found a strong cross-sectional relationship between religious involvement and lower BP. However, in longitudinal follow-up, the BP reduction was not significant.^39^

On the other hand, the Black Women’s Health Study, a large cohort study that followed up 59,000 African-American women for 23 years, found that the intense religious/spiritual involvement when coping with stressful events was associated with a lower risk of developing AH than no involvement. The association was stronger among women who reported higher levels of perceived stress. However, frequent prayer was associated with a higher risk of AH.^33^

A Brazilian study found that the prevalence of AH in highly religious communities was lower than the national average.^40^ Holt-Lunstad et al. monitored ambulatory blood pressure in a cohort of 100 outpatients, finding that a high level of spirituality was associated with lower BP values.^41^

The NHANES III survey collected data on BP, reported frequency of attendance at religious services, and history of AH treatment from 14,475 American men and women aged 20 years or older. Compared to participants who never participated, the systolic BP of those who attended weekly services was 1.46 mmHg lower (95% CI, 2.33, 0.58 mmHg, p < 0.01), while that of participants who attended services more than 52 times/year was 3.03 mmHg lower (95% CI, 4.34, 1.72 mmHg, p < 0.01). No significant gender effect was observed, and these estimates were adjusted for a significant interaction between age and less-than-weekly frequency (1 to 51 times) (p < 0.05).^42^

Bell et al. studied the interrelationship between race/ethnicity, religious service attendance, and AH in non-Hispanic whites and blacks, and Mexican-Americans (N = 12,488). Compared with those who never attended religious services, whites who attended services weekly were less likely to have AH, as were blacks who attended more than once a week. However, there was no relationship between attending services and AH among Mexican-Americans, which suggests that these benefits are not the same for everyone.^43^

A cross-sectional study of 1,384 adult Tibetan Buddhists from two Buddhist institutes in Sichuan Province, China also included 798 adults from nearby towns and cities. The risk of AH was significantly lower (38%) in Buddhists than non-Buddhists. In a subgroup of 570 Buddhists, longer participation in Buddhist activities was associated with a lower prevalence AH, as well as lower BP.^44^ In a longitudinal study, Timio et al. compared the BP of nuns in a secluded order with lay women over 30 years of follow-up. Although baseline data were similar for all variables, at the end of follow-up systolic and diastolic BP were significantly higher observed in the lay women.^45^ Thus, these studies suggest that religiosity/spirituality has a protective role in AH, which is mediated by behaviors that could be applied to the general population.^44^^,^^45^

In a study that examined hostility and the risk of cardiovascular disease (CVD) in young Hispanics, Sethness et al. found that aggressive feelings were associated with higher BP.^46^

In the Biopsychosocial Religion and Health Study (N = 9,581), lifestyle factors such as vegetarianism and regular exercise were important predictors of lower AH rates; intrinsic religiosity, even after controlling for these factors, was as strongly related to lower AH rates as lifestyle factors. This study demonstrated that, in addition to the positive effects of lifestyle choices, religion can have a direct positive effect on AH.^47^

The Nurses’ Health Study II evaluated 44,281 non-hypertensive women, observing that attendance at religious services had a modest inverse association with AH in a dose-response effect, ie, the higher the frequency, the lower the incidence of AH. The risk rate for those who attended more than once a week, compared to those who never or almost never attended, was 0.91 (95% CI, 0.86, 0.97).^48^

**Main messages**

**Table:** 

**Concepts**
Studies on the influence of psychological and religious/spiritual factors on high blood pressure have sought to determine how these factors participate in hypertension, but their results disagree.
In the prospective Nurses’ Health Study II trial, women who attended religious services were less likely to develop high blood pressure.
**Practical aspects**
Although studies do not completely agree about whether religiosity/spirituality results in a lower occurrence of arterial hypertension, some have found lower systolic blood pressure values and a lower incidence of arterial hypertension among practitioners, which was directly associated with the frequency of religious activity.

## High Blood Pressure, Spirituality and Other Psychosocial Determinants

Psychosocial factors must be considered in an individual’s risk profile. Prospectively following large samples with standard BP measures and psychosocial variables can establish the relationship between psychosocial factors and AH occurrence.^49^ However, although such epidemiological studies provide evidence about psychosocial influence, they do not provide data on pathophysiological mechanisms. Differences in the magnitude or pattern of reactions to behavioral stimuli could be related to the risk of developing AH.

Experimental studies can assess the impact of psychological stimuli under carefully controlled conditions. However, they have important limitations, since only acute responses are registered. A third type of investigation is assessing daily BP measurement in relation to psychosocial conditions and verifying their impact. The difficulty with this method is that daily BP is affected by a wide range of factors, such as exercise, diet, and other lifestyle habits. The model must receive adequate statistical treatment to distinguish the influence of psychosocial factors.^49^ Thus, different study types have both strengths and limitations. To assess the influence of psychosocial factors, it is necessary to integrate different approaches.

### Psychosocial Characteristics

A number of psychological characteristics have been associated with AH, including anxiety, anger, hostility, mental health, social problems, work-related difficulties, and depression.^49^^,^^50^ However, the most consistent evidence is related to anger and hostility traits.^49^

Anger-related traits may not be apparent all the time, but they do reveal themselves when people are confronted or feel threatened. Anger is considered relevant to the development of AH or in individuals who experience chronic stress due to their living or work conditions. Using arterial blood pressure monitoring, Casagrande et al. demonstrated that sustained feelings of anger contribute to a lack of nocturnal decline in blood pressure, which is related to a worse prognosis.^51^

In another study, 60 boys aged 12-16 assigned to challenging mental tasks were evaluated for physiological and subjective measures before and after the task.^52^ The boys, who were classified as having or not having a disposition towards anger inhibition, did not differ in resting conditions. However, in response to the task, boys who reported high levels of anger inhibition had a greater systolic blood pressure response. Interestingly, this was the pattern among participants with a family history of AH, which suggests that genetic factors also interact with psychological traits and situational dispositions that can influence the risk of AH.

### Study Characteristics

Studies have shown that certain types of work are associated with AH risk. The highest risk is when the demands of the job outweigh the rewards.^49^ A case-control study involving 30- to 60-year-old men working at different locations in New York showed that job stress was an independent determinant of AH after controlling for age, body mass index, type A personality, 24-hour urinary excretion of sodium, physical activity at work, education, smoking, and alcohol consumption.^53^ After 3 years, those with persistently high job stress levels had higher BP. This response may be related to the rhythm of task performance and its relationship with baroreflex suppression when the demand level is minimal.^49^

Job insecurity can generate anger and psychological ailments such as that arising from working in companies with high layoff rates cause apprehension among employees.^50^ Interestingly, workload does not seem related to higher BP.^50^

The CARDIA study, which evaluated 8,395 white-collar workers in Canada, looked at both cumulative exposure and new workload. After 7.5 years of follow-up, higher BP was observed among workers with low social support levels at work.^54^ An observational study also found that a considerable pay raise can reduce the risk of hypertension by 16%.^50^

### Isolation and Social Support

Social support helps us deal with life and its adversities, while isolation, defined in terms of the size and composition of the social network (eg, marital status, the number of friends and close relatives, and religious or other groups) has been associated with cardiovascular disease and mortality.^49^ Isolation or low social support is associated with higher BP.^49^ Social relationships are important sources of emotional and practical support and can moderate the psychological effects of stress. Lack of support and relationships not only leaves a person without these resources, but can be a major source of stress.

Among the various elements of the social network, marriage is often the central relationship in people’s lives. Married individuals tend to have better health outcomes than those who live alone, while other sources of support do not fully offset the effects of loneliness. However, relationships can also be a source of conflict, and the stress associated with unhappy or strained marriages has been associated with harmful cardiovascular effects. Acute episodes of marital conflict have been shown to induce elevations in BP.^54^

### Mental Health

Patients who re-experience symptoms related to a previous trauma have significantly higher rates of AH.^50^ One study found that perceived stress can be considered a risk factor for AH in women with low-income occupations. Studies that address the relationship between stress and AH should highlight possible interactions between sex and occupational status.^55^

Suffering racism can adversely affect health; data from the Black Women’s Health Study indicated that there may be an association between experiences of racism and an increased incidence of AH.^56^

Acute stress promotes a transient increase in BP, but there is no consistent evidence that this effect results in sustained AH. Chronic stress and, especially, a maladaptive stress response are likely involved in the development of AH.^57^ A meta-analysis of cohort studies evaluated the effect of emotional stress on BP, finding that a BP increase was 21% more likely in individuals who had more intense responses to stressful tasks (odds ratio [OR]: 1.21; 95% CI, 1.14-1.28; p < 0.001). Although the effect magnitude was small, the results suggest the relevance of controlling psychological stress to prevent increased BP.^58^

### Depression and Sleep Disorders

The association between depression and the risk of developing AH has been assessed in two studies, whose results were inconsistent. In the Whitehall cohort study, participants in the “increasing depression” group (characterized by an increase in depressive episodes over the years) had a 25% lower risk of AH at 35 to 39 years of age compared to the “low/transient depression” group. However, there was a faster age-related increase in AH in the “increasing depression” group, corresponding to a 7% higher risk of AH with each 5-year increase in age. Thus, the risk of AH in participants in the “rising depression” group at the end of follow-up was substantially higher than in the “low/transient depression” group, and this pattern was more significant in men than in women. This study suggests that the risk of AH increases with repeated depressive episodes and becomes evident in later adulthood.^59^

NHANES I 1982-1992^60^ examined the effects of sleep duration and insomnia on the association between depression and AH. However, as in the previous study, the results depended on the age of the cohort. Depressed individuals aged 32 to 59 years had a 44% higher risk of AH in the 10-year follow-up (OR: 1.44, CI: 1.15-1.80). Individuals who reported sleeping 5 hours or less per night were 50% more likely to develop AH than those who slept 7 to 8 hours per night (OR: 1.50, CI: 1.11-2.02). There was no association between depression, sleep quality, and AH in participants aged 60 to 86 years.

All studies show a direct relationship between sleep disturbances and the incidence of AH. In the Penn State Cohort, the risk of AH in people with short sleep duration or sleep disorders was significantly higher, but it became marginally significant after controlling for obesity (OR: 1.6, 95% CI, 0.9- 2.8). Chronic insomnia is associated with an increased risk of AH. Thus, it is possible that sleep duration is a determinant of AH.^61^

### Personality

There are controversial results about the relationship between personality and AH risk. The type D personality, characterized by high levels of negative affectivity and high inhibition, was not found to be associated with elevated systolic and/or diastolic blood pressure. However, lower levels of awareness, characterized by disorganization, irresponsibility, indiscipline, negative emotions, and overreaction to stress have been associated with AH.^50^

**Main messages**

**Table:** 

**Concepts**
Psychosocial factors that affect BP behavior and, thus, in AH onset include: personality, type of work, social isolation, mental health, depression, and sleep disorders
**Practical aspects**
These conditions must be assessed to determine when they can interfere in BP behavior and AH control.

## Spirituality in Blood Pressure Treatment Adherence

Beside non-drug treatments, AH is treated with antihypertensive drugs of recognized efficacy and effectiveness.^3^^,^^29^^,^^62^ Controlling BP modifies the natural history of the disease, reducing morbidity and mortality. However, a barrier to this objective in patients worldwide is lack of adherence to lifestyle changes or prescribed medications.^3^^,^^29^^,^^62^ Religiosity/spirituality has been recognized as a cultural aspect that interferes with the attitudes of individuals.^63^^–^^68^

For more than 4 decades, countless studies have found that spirituality, which in its broadest sense permeates aspects of religiosity itself, but mainly promotes self-knowledge, self-confidence, resilience, belief in the possibility of self-determination, and a positive vision of the world and the future, is associated with better adherence to the advice of health professionals.^63^^,^^66^^,^^69^^,^^70^

Spirituality has a strong influence on certain lifestyle habits that are directly related to hypertension and the quality of life of hypertensive patients.^31^^,^^71^ Several clinical studies have shown that transcendental meditation (TM) helps reduce BP in hypertensive patients.^72^^,^^73^ A meta-analysis of these randomized studies showed that TM reduces BP between 4 and 6 mmHg in a dose-dependent manner (ie, more sessions lead to greater BP reduction).^74^

A major clinical trial randomized 201 African-Americans with coronary artery disease to either a TM or a health education program. In a mean follow-up of 5 years, there was a 48% risk reduction in the primary outcome of death, stroke, or acute myocardial infarction (OR: 0.52, 95% CI, 0.29-0.92, p = 0.025). There was also a reduction in mean systolic BP (5 mmHg), as well as anger expression according to specific scales (p < 0.05). The study’s conclusion was that TM can be beneficial in the AH management and in secondary prevention of stroke.^75^

Yoga-based interventions have been studied in CVD management. A meta-analysis of 17 studies showed that yoga was associated with a significant reduction in systolic and diastolic blood pressure, especially if all three elements of yoga are used (breathing, posture, and meditation).^76^

Although evidence about the direct impact of religious/spiritual-based interventions on AH is less robust, a significant amount of data has shown a beneficial association between religious activity (religious affiliation, organized or private religious services) or spiritual characteristics (tendency toward optimism, gratitude, and positive emotions) and a lower incidence of AH or better BP control in already hypertensive individuals.^77^^,^^78^

The Black Women’s Health Study found that religiosity/spirituality helps them cope with everyday life situations and contributes to BP control.^33^

Although the relationships between religiosity/spirituality and adherence to drug treatment in chronic diseases may vary according to the population, in general, it has a positive influence.^1^^,^^79^^–^^82^ Spirituality can lead to improved adherence to medications through better control of negative feelings, such as anxiety, stress and depression.^65^

Small qualitative studies have found that African-American women believe that spirituality helped them adhere to drug treatment.^83^^,^^84^ On the other hand, in Ghanaian women, religiosity/spirituality was associated with worse adherence to AH treatment due to the hope of a divine cure.^85^

Other studies have found no correlation between religiosity/spirituality and treatment adhesion, not even considering patient trust in the physician. However, trust in the physician has been associated with greater adherence to antihypertensive medications.^67^^,^^86^ Other factors may also influence treatment adherence, such as self-efficacy, cultural beliefs and knowledge about the disease.^87^

Health professionals must understand the importance of religiosity/spirituality, recognize and value cultural aspects, and respect their patients’ beliefs and hopes. This apparently simple attitude can provide new opportunities for successful treatment of chronic disease patients, since such conditions require greater discipline, perseverance, and faith.

**Main messages**

**Table:** 

**Concepts**
A significant amount of data indicates a beneficial association between religious (religious affiliation, organized or private religious services) or spiritual aspects (tendency toward optimism, gratitude, and positive emotions) and a lower incidence of AH or better BP control in already hypertensive individuals It has been demonstrated for over 4 decades that religiosity, self-knowledge, self-confidence, resilience, belief in the possibility of self-determination, and a positive vision of the world and the future, is associated with better adherence to the advice of health professionals
**Practical aspects**
Health professionals must understand the importance of religiosity/spirituality, recognize and value cultural aspects and respect their patients’ beliefs and hopes. This apparently simple attitude can provide new opportunities for successful treatment of chronic disease patients, since such conditions require greater discipline, perseverance, and faith.

## Spirituality and Relevant Cardiovascular Events

Observational studies have been investigating the association between spirituality, religiosity, and clinically relevant outcomes in different areas of health (especially CVD) since the 1980s.^88^

The InterHeart study evaluated risk factors independently associated with acute myocardial infarction in 52 countries, including Brazil, finding that 90% of population attributable risk was associated with nine modifiable risk factors: dyslipidemia, smoking, abdominal obesity, diabetes, hypertension, sedentarism, unhealthy diet, alcohol use, and psychosocial factors. Psychosocial risk factors (stress and depression) accounted for 33% of the population attributable risk for acute myocardial infarction, an impact greater than that of established risk factors such as hypertension and diabetes.^89^ In addition, InterStroke assessed independent risk factors for stroke. Following the same InterHeart strategy, the study found that stress and depression were responsible for 17% of the population attributable risk for stroke worldwide.^90^

In 2000, a meta-analysis of 42 studies, including approximately 126,000 individuals, found that religious involvement was associated with a lower all-cause mortality rate.^91^ Subsequent studies have shown an inverse association between mortality and frequency of attendance at religious services, and that this association was substantially mediated by health behaviors and other risk factors.^42^^,^^92^

Two large cohort studies published in 2016 and 2017 reinforced these findings. In the Black Women’s Health Study, which included 36,613 African-American women, the mortality rate was 46% lower among those who attended religious services several times a week than among those who did not attend. There was no association between daily prayer, religious confrontation, or self-identification as a very religious/spiritual person with mortality.^93^

The Nurses’ Health Study, which followed up more than 74,000 nurses for up to 8 years, found that attendance at religious services at least once a week was associated with 33% lower all-cause mortality, as well as substantially lower mortality from CVD and cancer, than not attending.^94^

On the other hand, in a cohort of 2,068 acute coronary syndrome patients, there was no association between comfort from religion, prayers for their own health, or the prayers of others on their behalf, and survival in up to 2 years of follow-up.^95^ However, patients who prayed for their own health and those who were aware that others were interceding for them experienced improved health-related quality of life over time.^96^ The Multiethnic Study of Atherosclerosis found no consistent patterns of association between measures of religiosity and the presence/extent of subclinical CVD at baseline or CVD incidents over 4 years.^97^ The findings are conflicting about the association between religiosity/spirituality and CV risk factors. The Women’s Health Initiative study, which included more than 43,000 menopausal women, found a higher CV risk in patients with private spiritual activity, such as prayer, Bible reading, or meditation.^98^ In a Japanese cohort of 36,965 individuals, the more religious were significantly more likely to have better health habits and fewer CV risk factors, except for a higher prevalence of overweight/obesity at baseline. Religiosity was also associated with better health habits over time and less likelihood of developing diabetes, but not of developing AH or dyslipidemia.^99^

**Main messages**

**Table:** 

**Risk of coronary artery disease and aspects related to religiosity/spirituality**
In the InterHeart study, psychosocial risk factors (stress and depression) accounted for 33% of the population attributable risk for acute myocardial infarction.
**Risk of stroke and aspects related to religiosity/spirituality**
In the InterStroke study, psychosocial factors (stress and depression) accounted for 17% of the population attributable risk for stroke.
**Practical aspects**
Although the results do not agree regarding the benefits of religiosity/spirituality on cardiovascular health, some studies have shown a reduction in cardiovascular events and mortality in those individuals with religious/spiritual practices.

## Perspectives, Knowledge Gaps and Conclusion

The concepts of evidence-based medicine have been gradually applied in the domain of spirituality and CVD research. Although the available scientific evidence can be considered promising, robust observational and randomized studies with adequate statistical power are still needed to confirm the benefits of spirituality in clinically relevant outcomes. Thus, scientific evidence about spirituality and CV medicine could influence and modify clinical practice and patient prognosis.

For strength and robustness, this position paper sought data on AH and spirituality from diverse areas of research. Health professionals must approach the spirituality of their patients according to well-defined criteria for action.

As a strictly evidence-based review, we did not find conclusive evidence that religiosity/spirituality is “a safe and useful/effective procedure” (ie, data obtained from several large studies and concordant and/or robust meta-analysis of randomized clinical trials: grade of recommendation I and level of evidence A). However, neither did we find evidence and/or consensus that “the procedure is not useful/effective and, in some cases, harmful” (ie, grade of recommendation III and level of evidence C).

What is important is these concepts are implemented with scientific support, offering physicians a way to expand their performance and offer patients resources for dealing with disease, illness, and life in addition to indispensable traditional medical approaches.

Thus, it is our duty to offer the fullest sense of health, ie, physical, social, emotional, psychological, and spiritual well-being, applying spiritual concepts sparingly and sensitively to the patients entrusted to our care.
